# Two ancient membrane pores mediate mitochondrial-nucleus membrane contact sites

**DOI:** 10.1083/jcb.202304075

**Published:** 2024-03-08

**Authors:** Jana Ovciarikova, Shikha Shikha, Alice Lacombe, Flavie Courjol, Rosalind McCrone, Wasim Hussain, Andrew Maclean, Leandro Lemgruber, Erica S. Martins-Duarte, Mathieu Gissot, Lilach Sheiner

**Affiliations:** 1 Wellcome Centre for Integrative Parasitology, University of Glasgow, Glasgow, UK; 2 CNRS, Inserm, CHU Lille, Institut Pasteur de Lille, U1019—UMR 9017—CIIL—Center for Infection and Immunity of Lille, University of Lille, Lille, France; 3 Departamento de Parasitologia, Instituto de Ciências Biológicas, Universidade Federal de Minas Gerais, Belo Horizonte, Brazil

## Abstract

Coordination between nucleus and mitochondria is essential for cell survival, and thus numerous communication routes have been established between these two organelles over eukaryotic cell evolution. One route for organelle communication is via membrane contact sites, functional appositions formed by molecular tethers. We describe a novel nuclear-mitochondrial membrane contact site in the protozoan *Toxoplasma gondii*. We have identified specific contacts occurring at the nuclear pore and demonstrated an interaction between components of the nuclear pore and the mitochondrial protein translocon, highlighting them as molecular tethers. Genetic disruption of the nuclear pore or the TOM translocon components, TgNup503 or TgTom40, respectively, result in contact site reduction, supporting their potential involvement in this tether. TgNup503 depletion further leads to specific mitochondrial morphology and functional defects, supporting a role for nuclear-mitochondrial contacts in mediating their communication. The discovery of a contact formed through interaction between two ancient mitochondrial and nuclear complexes sets the ground for better understanding of mitochondrial-nuclear crosstalk in eukaryotes.

## Teaser

By studying a single-cell parasite, this work reveals a new contact between the energy and metabolism hub of the cell, the mitochondrion, and the organelle that hosts the cell genome, the nucleus. The contact is likely mediated by abundant and ubiquitous pores in their membranes.

## Introduction

Organelles provide the essential compartmentalization that is required to delineate distinct biochemical environments within the eukaryotic cell. However, eukaryotic organelles are also highly interconnected, and this is necessary for critical communications between different cellular pathways hosted in distinct compartments. Membrane contact sites are functional appositions between organelles that mediate this essential connectivity. Tether molecules that reside in each of the membranes in contact mediate their apposition, bringing the two organelles within a short distance from each other, ranging between 10 and 80 nm (Scorrano et al., 2019), thus enabling the efficient and controlled transfer of signals and metabolites. These contacts exist between any two organelles studied to date, and they are assigned multiple important functions including phospholipid biogenesis, calcium homeostasis, control of autophagy and of organelle fission, and inheritance (Gatta and Levine, 2017; Zung and Schuldiner, 2020). Membrane contact sites are thus central to the structure and function of any eukaryotic cell, and it is therefore important to understand their composition and function in a variety of divergent eukaryotes (Santos and Nozaki, 2021; Ovciarikova et al., 2022a).

As the host of the genome, the cell nucleus plays a critical role in the control of all cellular functions. No less important, the mitochondrion is the major producer of energy and essential metabolites and cofactors in the cell. Therefore, it is easy to understand why the communication and coordination between these two central organelles are imperative for cell survival. On one hand, mitochondrial functions rely almost entirely on the expression of genes encoded in the nuclear genome, and thus any mitochondrial changes must be communicated to the nucleus to adjust gene expression (Quirós et al., 2016). On the other hand, mitochondrially produced metabolites are necessary for nuclear functions such as epigenetics modification and genome replication (Walker and Moraes, 2022). Indeed, different modes of communication between the mitochondrion and the nucleus have evolved to support cell survival, and this has been studied extensively (Eisenberg-Bord and Schuldiner, 2017; Walker and Moraes, 2022). Yet the role of contact sites in facilitating this communication is only starting to be explored.

The first nuclear mitochondrial tether was reported recently in a study that combined fluorescence maker of nuclear-mitochondrial proximity and two library screens in yeast (Eisenberg-Bord et al., 2021). The study identified the nuclear envelop protein named contact nucleus mitochondria 1 (Cnm1) as a tether component and showed that it interacts with the mitochondrial protein import component Tom70 to tether the two organelles (Eisenberg-Bord et al., 2021). The finding of a component of the translocon of the outer mitochondrial membrane (TOM) complex in a mitochondrial membrane contact site is consistent with several previous observations whereby TOM takes part in forming contacts (Elbaz-Alon et al., 2015; Murley et al., 2015; González Montoro et al., 2018; Namba, 2019). A contact site between the nucleus and mitochondria was also recently described in human cells. The study hypothesized that a nuclear-mitochondrial contact might catalyze mitochondrial retrograde response (MRR) as part of prosurvival pathways in cancer cells (Desai et al., 2020). The study provided evidence for the cholesterol-binding mitochondrial outer membrane translocator protein (TSPO) as a key candidate for tethering. The study further identifies the A-kinase anchoring protein acyl–coenzyme A binding domain containing 3 (ACBD3), the protein kinase A (PKA), and the A-kinase-anchoring protein, AKAP95, as the potential partners linking TSPO to the nucleus (Desai et al., 2020).

Apicomplexan parasites are single-cell organisms that belong to a distinct branch of the eukaryotic tree (Discoba) than yeast and humans (Ophistokont) and are highly divergent from those common model systems. Still, nuclear-mitochondrial communication is expected to be critical for survival in these organisms just the same. For example, in the malaria-causing Apicomplexa, *Plasmodium falciparum*, enhanced mitochondrial-nuclear proximity is seen in parasites that persist after treatment with the antimalarial dihydroartemisinin (DHA) (Connelly et al., 2021). Mitochondrial-nuclear communication was thus proposed to mediate prosurvival MRR as in cancer cells (Connelly et al., 2021); however, the observed proximity is within a distance larger than most membrane contacts described to date, and a tether has not been identified. Moreover, homologs of TSPO or Cnm1 and Tom70 are not found in the apicomplexan genomes. Hence, it became important to explore whether nuclear mitochondrial contact sites exist in these organisms and what are the molecular tethers.

Here, we describe a contact between the nuclear envelope and the mitochondrion in the model apicomplexan *Toxoplasma gondii*. We find that some of the observed nuclear mitochondrial contacts occur near the nuclear pore. We further uncover that an interaction between the nuclear pore and the TOM complex in the mitochondrial outer member likely tethers this contact and that depletion of nuclear pore component TgNup503, or the TOM component TgTom40 leads to a reduction in nuclear mitochondrial contacts, pointing at their potential involvement in tethering. Finally, we show that depletion of TgNup503 results in mitochondrial morphological defects coupled with a mild enhancement of mitochondrial membrane potential. Our study paves the way for a more comprehensive understanding of nuclear–mitochondrial communication in divergent eukaryotes.

## Results

### Mitochondria and nucleus membrane contact sites are observed in *Toxoplasma*, with some encompassing the nuclear pore

**Figure 1.**
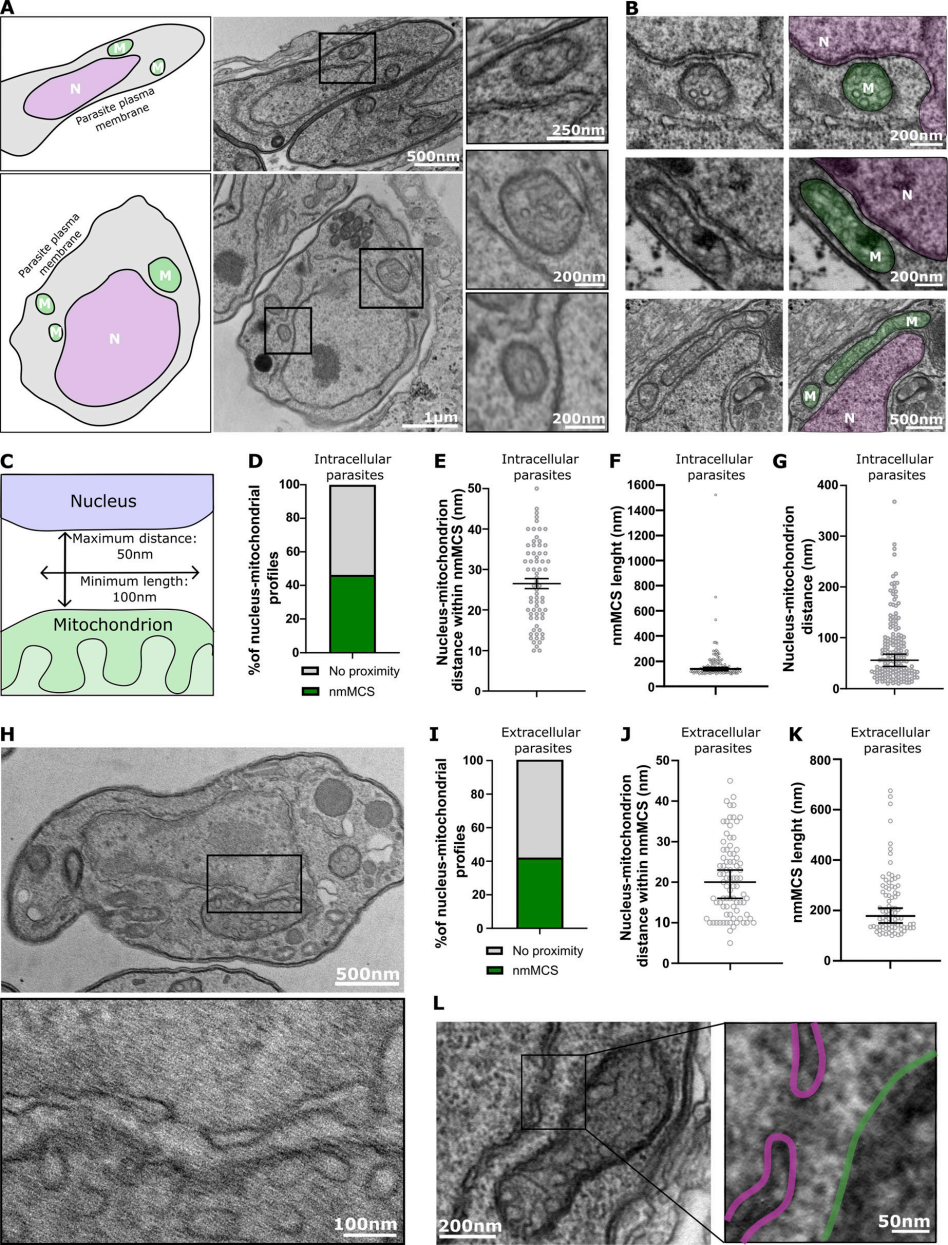
**Characterization of mitochondrion-nucleus contact sites and proximity between the nuclear pore and mitochondrial outer membrane in contacts detected by EM. (A)** EM images of *T. gondii* displaying a section of entire parasites whereby areas of close apposition between the single mitochondrion and the nucleus are marked with boxes with closeup of the boxed region shown to the right. Schemes illustrate the full parasites and highlight the two organelles: nucleus (N) and mitochondrion (M). **(B)** Additional EM images of close appositions representing the range of lengths of contacts observed and with colored mitochondrion (green) and nucleus (pink). **(C)** Scheme of the criteria used to define a contact. A membrane contact site (MCS) is defined as a maximum distance of 50 nm between the membranes that extend for a minimum of 100 nm. **(D–F)** Quantification of the characteristics of the observed contacts in the EM images. The abundance (two biological replicates, 134 events in total) of contacts (D); distances between the membranes (from two biological replicates, 68 profiles) (E); and lengths of contacts (four independent experiments, performed with different *T. gondii* lines, 132 events in total) (F) were assessed. Error bars for distance within nmMCS display mean with SEM. Error bars for average length display median with 95% confidence intervals. **(G)** Quantification of all the distances seen between nuclear and mitochondria in 177 profiles that contain both organelles collected from two independent experiments. Error bars display median with 95% confidence intervals. **(H)** EM image of extracellular *T. gondii* displaying a section of entire parasites whereby areas of close apposition between the single mitochondrion and the nucleus are marked with boxes with closeup of the boxed region shown below. **(I–K)** Quantification of the characteristics of the observed contacts in EM images from extracellular parasites: abundance (H, from four biological replicates, 198 profiles); distances (I) and lengths (J) both from four biological replicates, 83 profiles). Error bars for average length display median with 95% confidence intervals. **(L)** An image showing a nuclear pore within the observed contact. The mitochondrial membrane is highlighted in green and nuclear membrane in pink.

We first explored the occurrence of nuclear-mitochondrial membrane contact sites in *Toxoplasma gondii* tachyzoites through the analysis of EM images (Fig. 1, A and B). We followed the criteria that are defined by the field (Scorrano et al., 2019), which we previously used to define ER-mitochondria and mitochondria-pellicle contact sites in *Toxoplasma* via EM (Ovciarikova et al., 2017; Mallo et al., 2021). We identified regions of juxtaposition between the nuclear envelope and the mitochondrial outer membrane where the proximity is 50 nm or less and where the stretch of proximity continues over 100 nm or more (Fig. 1 C). Among the 134 parasite EM profiles where both nucleus and mitochondria could be observed, we detected that 46.3% of mitochondria form structures corresponding to our criteria (Fig. 1 D). The distances in those contacts varied from 10 to 50 nm, which we defined as the cut-off, with a median of 26 nm (Fig. 1 E). The length of those contacts varied from 100 nm, which we defined as shortest stretch, to 1,523 nm, which was the longest contact detected, and the median length is 138.8 nm long (Fig. 1 F). For comparison, when we analyzed a total of 177 profiles showing both organelles and measured all distances between them (not focusing only on contacts), we found that the distance ranges from 10 to 368 nm with median of 56 nm (Fig. 1 G).

We previously described a drastic change in mitochondrial morphology when *Toxoplasma* parasites become extracellular (Ovciarikova et al., 2017). We therefore analyzed nuclear mitochondrial contacts in extracellular parasites too (Fig. 1 H). Among the 198 EM profiles where both organelles could be observed, we detected that 41.9% of mitochondria form structures corresponding to our criteria for contact (Fig. 1 I). The distances in those contacts varied from 5 to 45 nm, with a median of 20 nm (Fig. 1 J). The length of those contacts varied from our defined shortest stretch of 100–675 nm, and the median length was 178 nm long (Fig. 1 K).

**Figure S1.**
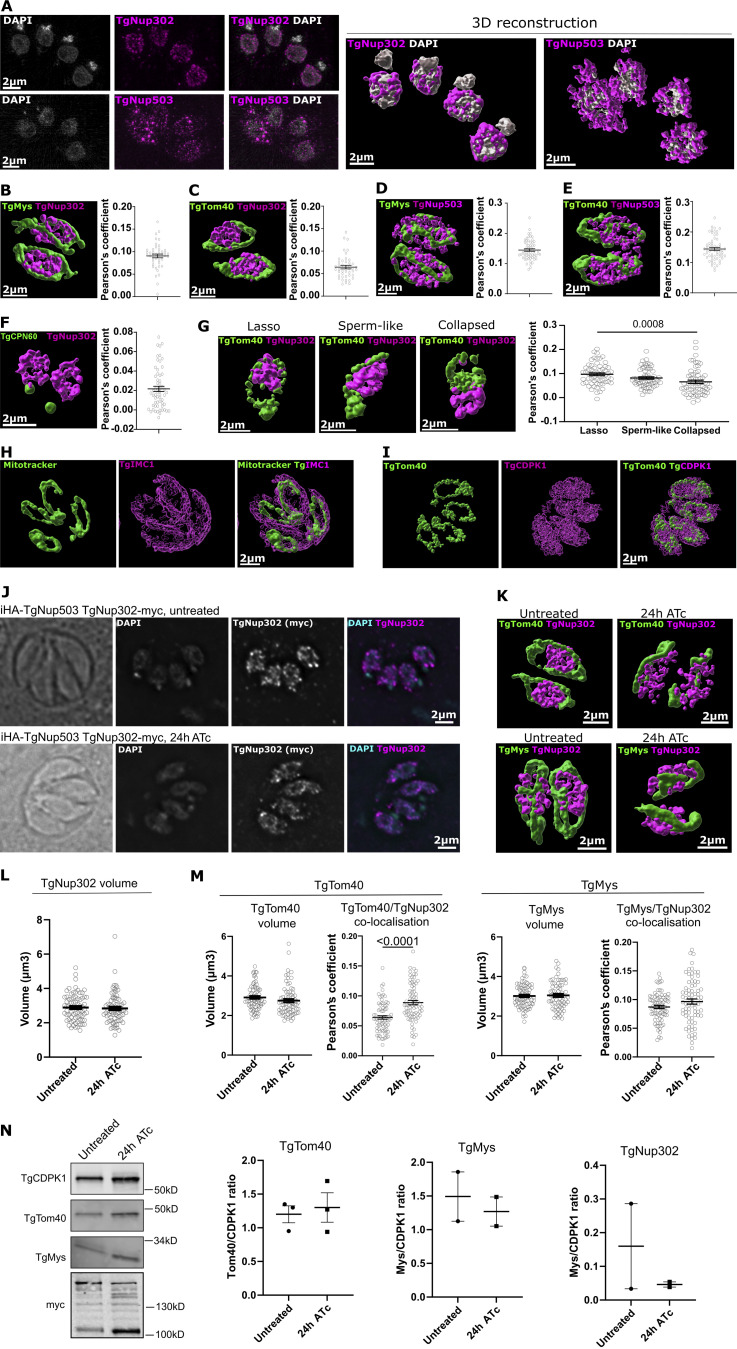
**Super-resolution microscopy analysis of nuclear pore and outer mitochondrial membrane markers. (A)** Localization of the nuclear pore components TgNup302 and TgNup503 via super-resolution (SR) microscopy. A *T. gondii* line with endogenously tagged TgNup503 and TgNup302 (iHA-TgNup503 TgNup302-myc) was immunostained for the respective tags (pink) and costained with DAPI (grey). **(B–F)** The same line used in A (iHA-TgNup503 TgNup302-myc) was immunostained with a combination of markers, imaged with SR microscopy, and signal colocalization within a parasite vacuole (Pearson’s coefficient) was calculated: (B)—TgNup302 (anti-myc, pink) and anti-TgMys (green) (three biological replicates, 45 vacuoles in total); (C)—TgNup302 (anti-myc pink) and anti-TgTom40 (green) (three biological replicates, 44 vacuoles in total); (D)—TgNup503 (anti-HA, pink) and anti-TgMys (green) (three biological replicates, 60 vacuoles in total); (E)—TgNup503 (anti-HA, pink) and anti-TgTom40 (green) (three biological replicates, 60 vacuoles in total); (F)—TgNup302 (anti-myc pink) and anti-TgCPN60 (green) (three biological replicates, 59 vacuoles in total). Error bars display mean with SEM **(G)** TgNup302 (anti-myc, pink) and anti-TgTom40 (green) colocalization was assessed in extracellular parasites (iHA-TgNup503 TgNup302-myc line) where naturally lysed parasites were collected and attached to poly-L-lysine coated coverslips followed by immunostaining, SR microscopy, and calculation of colocalization (Pearson’s coefficient) for each mitochondrial morphology: lasso, sperm-like, and collapsed (three biological replicates, 60 parasites in total per mitochondrial morphology). Error bars display mean with SEM and data were analyzed with one-way ANOVA followed by Tukey’s multiple comparisons test. **(H and I)** *T. gondii* immunostained for mitotracker (green) and costained with the pellicle marker IMC1 (H) or the cytosolic marker CDPK1 (I) (pink). **(J)** Immunofluorescence detection of TgNup302 via anti-Myc antibody (pink) costained with the DNA dye DAPI (grey), shows that TgNup302 remains nuclear upon TgNup503 depletion (24 h ATc). **(K)** Representative 3D reconstruction of SR images used for the colocalization measurements in M, costained with anti-TgTom40 (top) and anti-TgMys (bottom) (both green) and TgNup302 (pink). **(L)** TgNup302 signal volume was measured in untreated and treated (24 h) parasites (iHA-TgNup503 TgNup302-myc) from 3D-reconstruction SR images. Each measurement represents the average TgNup302 volume per parasite within a vacuole (three biological replicates, 75 measurements total). Error bars display mean with SEM. Data were analyzed with two-sided unpaired *T* test (NS). **(M)** TgTom40 and TgMys signal volumes were measured in untreated and treated (24 h) parasites (iHA-TgNup503 TgNup302-myc) from 3D-reconstruction SR images (left graph for each marker). Each measurement represents the average volume per parasite within a vacuole (three biological replicates, 71–75 measurements total). TgTom40 and TgMys colocalization with TgNup302 (myc) was calculated (Pearson’s coefficient, three biological replicates with 75 measured vacuoles in total for each mitochondrial marker, right graph for each marker). Error bars display mean with SEM. Data were analyzed with two-sided unpaired *T* test. **(N)** Western blots showing levels of TgNup302 (myc), TgMys, and TgTom40 upon TgNup503 downregulation (24 h ATc) with TgCDPK1 as a loading control. The signal was quantified using secondary fluorescent antibodies. Two or three biological replicates were performed per protein. Error bars display mean with SEM. Data were analyzed with two-sided unpaired *T* test (NS). Source data are available for this figure: SourceData FS1.

Interestingly, we detected several cases where the observed contacts encompassed a structure characterized by discontinuation of the nuclear envelope corresponding to the nuclear pore (Fig. 1, H and L). To provide support and quantification for this curious observation, we first turned to analyze the proximity between the nuclear pore and mitochondrial outer membrane via fluorescent microscopy. Previous work identified some of the nuclear pore components in *Toxoplasma* (Courjol et al., 2017). TgNup302 is a putative homolog of yeast Nup145 that is autocatalytically cleaved, and its C-terminal part is found in the outer ring of the nuclear pore facing the cytoplasm. Additionally, TgNup503 interacts with TgNup302 in coimmunoprecipitation experiments (Courjol et al., 2017) and is a putative homolog of yeast Nup188/192, an inner ring component. Both proteins produce strong and stable signals by immunofluorescence when tagged, with TgNup302 tightly localizing around the nucleus, while TgNup503 shows a localization that spreads slightly beyond the nucleus (Courjol et al., 2017) (Fig. S1 A), potentially an outcome of the added endogenous tag. We utilized both tagged lines as nuclear pore markers to analyze their signal overlap with the signal from the mitochondrial outer membrane markers TgTom40 (van Dooren et al., 2016) and TgMys (Ovciarikova et al., 2017) in super-resolution microscopy. Pearson’s coefficiency value average of 0.064–0.1447 was found with all four marker combinations indicating overlap in the signal of both nuclear pore markers with both mitochondrial outer membrane markers (Fig. S1, B–E). For comparison, we measured signal overlap between the nuclear pore component TgNup302 and the parasite plastid marker CPN60 (Agrawal et al., 2009), which is expected to form only minimal interactions with the nucleus (Nishi et al., 2008). Pearson’s coefficiency value average for the plastid marker and nuclear pore component was 0.02167, substantially lower than the TgNup302 with either of the mitochondrial outer-membrane markers (Fig. S1 F). As mentioned above, mitochondrial morphology changes when *Toxoplasma* parasites become extracellular, where three main morphologies were defined: lasso, sperm-like, and collapsed (Ovciarikova et al., 2017). Using TgNup302 and TgTom40 as markers we analyze the proximity between the nuclear pore and the mitochondrion in extracellular parasites in all three shapes (Fig. S1 G). We observed proximity in all three forms with Pearson’s coefficiency values of 0.06544–0.09687, in the same range of the observations made in intracellular parasites, suggesting that the measured proximity is not dependent on the organelle shape and on the intracellular or extracellular state of the parasite. These observations provide preliminary support for a proximity between the nucleus and the mitochondrion at nuclear pore regions.

**Figure 2.**
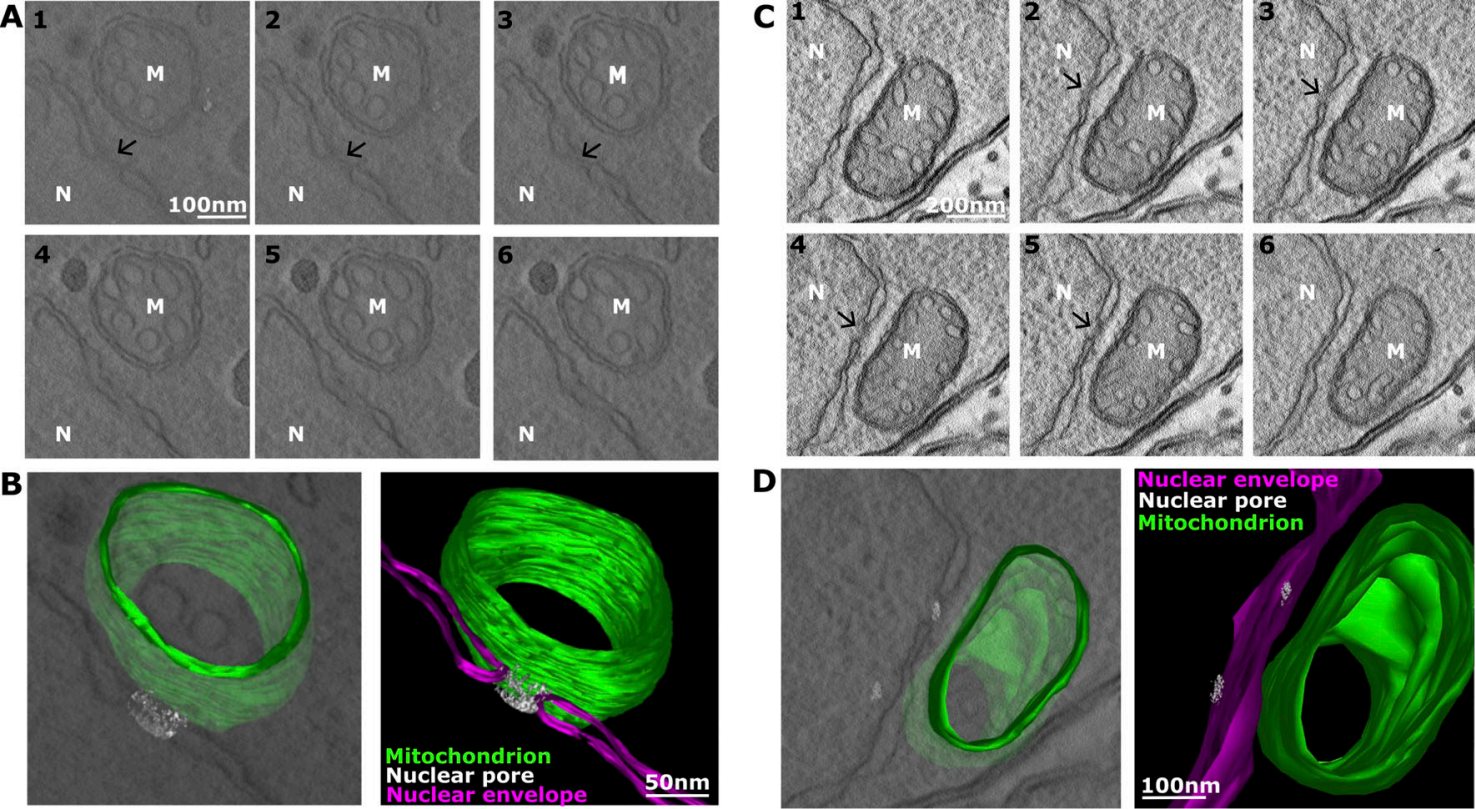
**EM tomography of two mitochondrion-nuclear contacts containing nuclear pores**. **(A–D)** Selected EM sections (A and C) and an image from the 3D reconstruction (B and D) of two contacts are shown; nucleus (N); nuclear membrane in pink; mitochondrion (M); mitochondrial membrane in green; nuclear pore is marked by an arrow and is also shown in grey.

However, the resolution obtained with the super-resolution microscopy method used is only sufficient to indicate proximity at 130–150 nm. Moreover, the large occupancy of the mitochondrion in intracellular tachyzoites (Fig. S1, H and I) limits the interpretation that can be drawn from the proximity observed. Thus, to characterize nuclear-mitochondrial contact sites that may involve the nuclear pore, we analyzed additional EM sections. We evaluated the occurrence of contacts that include a nuclear pore in 86 EM profiles where nuclear-mitochondrial contacts are seen collected from four independent EM experiments. We found that 13 (15.1%) of those contacts contained a nuclear pore. Finally, we performed EM tomography around two additional cases of nuclear mitochondrial contacts with a nuclear pore in them (one of which has two pores in the analyzed field). The tomography provides visualization of the contact in three dimensions from serial sections (Fig. 2) and shows that the contact occurs all around the nuclear pore (Videos 1 and 2). Together these observations demonstrate the occurrence of nuclear-mitochondrial membrane contact sites in *Toxoplasma* and the proximity of the nuclear pore and the mitochondrial outer membrane in some of those contacts.

**Video 1.** **3D reconstruction of two nuclear mitochondrial contacts that include the nuclear pore, seen in intracellular *T. gondii* tachyzoites.** The data were generated via electron tomography. A total of 242 images were acquired (from −60° to +60°, acquisition every 1°), with the reconstructed tomogram encompassing a total of 250 nm microns depth. The z-slices are shown in grey, and then mitochondria (green), nuclear envelope (magenta), and nuclear pore (light grey) are highlighted and shown from different directions, with zoom-in on one of the two contacts. Scale bar = 200 nm.

**Video 2.** **3D reconstruction of a nuclear mitochondrial contact that included two nuclear pores, seen in intracellular *T. gondii* tachyzoites.** The data were generated via electron tomography. A total of 242 images were acquired (from −60° to +60°, acquisition every 1°), with the reconstructed tomogram encompassing a total of 250 nm microns depth. The z-slices are shown in grey, and then mitochondria (green), nuclear envelope (magenta), and nuclear pore (light grey) are highlighted and shown from different directions, with zoom-in on one of the two contacts. Scale bar = 200 nm.

### Nuclear pore proteins interact with the mitochondrial outer membrane translocon component, TgTom40

**Figure S2.**
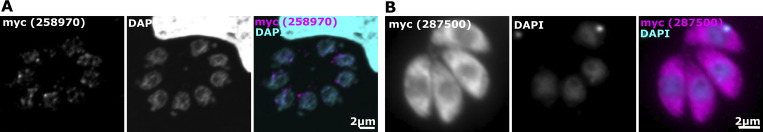
**Immunofluorescence of two hypothetical proteins found in the pull-down of TgNup503. (A)** TGGT1_258970 tagged with Myc detected with anti-Myc (pink) and costained with the DNA dye DAPI. **(B)** TGGT1_287500 tagged with Myc detected with anti-Myc (pink) and costained with the DNA dye DAPI.

The presence of nuclear pores in some of the observed contacts led us to hypothesize that nuclear pore components may mediate them. As a first step to test this hypothesis, we utilized the tagged TgNup503 for a pull-down experiment to identify what proteins it may interact with. Using the N-terminally HA-tagged TgNup503, we performed two repeats of pull-down experiments, alongside three repeats of negative control, where we performed the pull-down using the anti-HA antibodies from a parental line where no protein was HA-tagged. We recovered 10 proteins that were pulled down in both repeats with more than five peptides identified and that are not seen in any of the three controls (Table 1 and Table S1). As expected, other nuclear pore proteins (TgNup302, TgNup129, and TgNup593) were identified among the interactions recovered, providing validation to the pull-down experiment. In addition, two hypothetical proteins were recovered, TGGT1_258970 and TGGT1_287500, for which tagging and immunofluorescence revealed nuclear and cytosolic localizations, respectively (Fig. S2, A and B).

**Table 1.** Interactions of TgNup503 identified via pull down

ToxoDB gene id	Protein name	Peptide number found in each pull down repeat				
		Pull down with TgNup503		Negative control		
		R1	R2	NR 1	NR 2	NR 3
TGME49_269290	TgNup503	172	169	0	0	0
TGME49_258970	Hypothetical protein	11	17	0	0	0
TGME49_208430	Serine proteinase inhibitor PI-2 putative	6	8	0	0	0
TGME49_287500	Hypothetical protein	5	4	0	0	0
TGGT1_276890	TgNup129	4	10	0	0	0
TGGT1_220400	Nucleoporin autopeptidase	4	5	0	0	0
TGME49_218280	TgTOM40	4	5	0	0	0
TGGT1_313430	TgNup593	3	7	0	0	0
TGME49_259640	TgNup302	2	5	0	0	0

The full table of genes retrieved in the pull-down experiments is in Table S1. Here we summaries the hits that were found in both pull-down repeats (R1, R2) and not in the three negative control repeats (NR 1–3) and for which five peptide or more were found in at least one of the two repeats.

**Figure S3.**
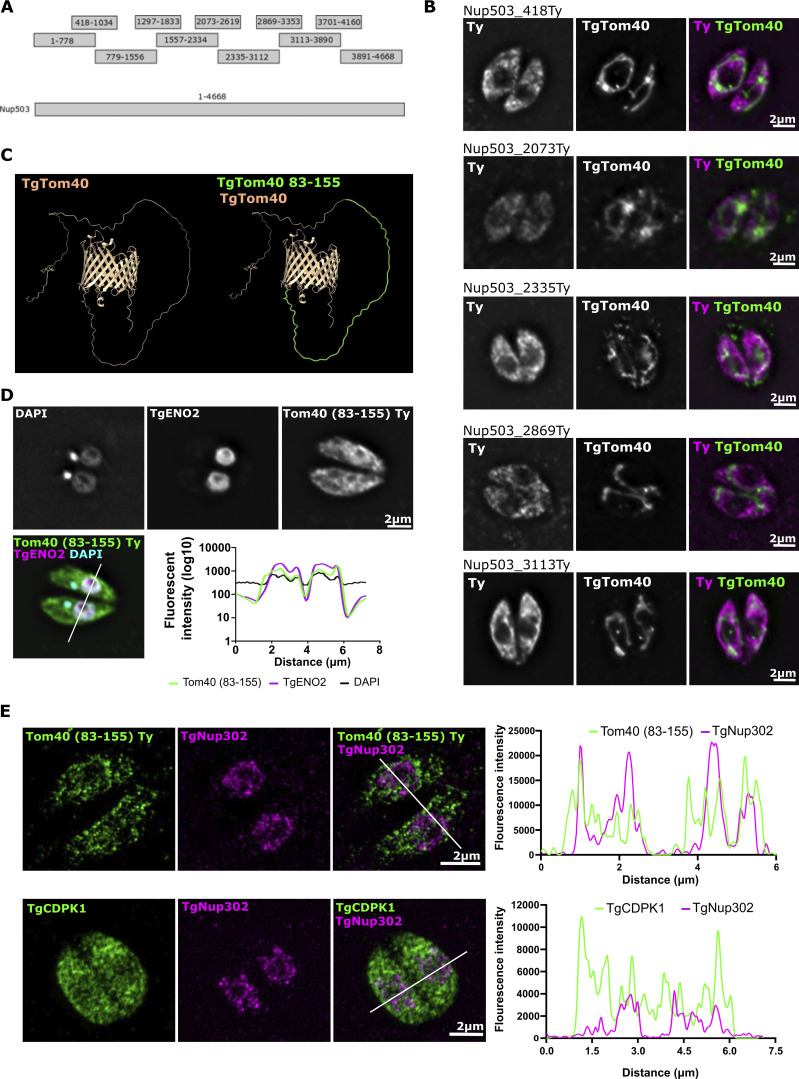
**Attempts to express fragments of TgNup503 or TgTom40, or to overexpress TgNup503, resulted in the localization a TgTOM40 fragment to the nuclear periphery. (A)** A scheme showing the TgNup503 fragments attempted to test for localization. **(B)** Transient expression of five TgNup503 fragments seen in A C-terminally Ty tagged and immunofluorescence with anti-Ty (Pink) and the mitochondrial marker TgMys (green). **(C)** AlphaFold model for *T. gondii* Tom40. TgTom40 fragment 83–155 highlighted in green. **(D)** Transient expression of TgTom40_83–155 C-terminally Ty tagged fragment, and immunofluorescence with anti-Ty (green) and two nuclear markers the DNA staining DAPI (grey) and anti-TgENO2 (pink). Signal quantification for DAPI, anti-TgENO2, and TgTom40 fragment (anti-Ty) in a parasite cross-section. **(E)** Transient expression of TgTom40_83–155 C-terminally Ty-tagged fragment into a line with Myc-tagged TgNup302, and immunofluorescence with anti-Ty (green) and anti-Myc (pink) was performed using super-resolution microscopy. As a control, immunofluorescence with anti-TgCDPK1 (green) and anti-Myc (pink) was done. Signal quantification for TgNup302 and TgTom40 fragment (anti-Ty) or TgCDPK1 in a parasite cross-section.

Among the potential interactions recovered in the pull-down, we observed the mitochondrial outer membrane protein translocon component TgTom40 (Table 1 and Table S1). This caught our attention both due to the suitable location of TgTom40 in the outer mitochondrial member where it could mediate this contact and because components of the TOM translocon are involved in several other mitochondrial membrane contact sites in various organisms (Lahiri et al., 2014; Elbaz-Alon et al., 2015; Murley et al., 2015; González Montoro et al., 2018; Namba 2019; Eisenberg-Bord et al., 2021). We reasoned that if epitopes of TgNup503 are involved in this interaction with TgTom40, then if we express fragments of TgNup503, any fragment that includes the amino acids involved in the interaction would localize around the mitochondrion. This has been seen in yeast upon expressing fragments of the nuclear Cnm1 which interacts with Tom70 (Eisenberg-Bord et al., 2021). However, the transient expression of each of the five fragments of TgNup503 resulted in cytosolic localization with no clear accumulation around the mitochondrion (Fig. S3, A and B), and we were unable to express the remaining six fragments. We thus attempted the reciprocal approach. We expressed a long fragment of TgTom40, TgTom40^83–155^, that is expected to be accessible for interactions based on structural prediction via α-fold (Fig. S3 C) and examined if it accumulates around the nucleus. TgTom40^83–155^ showed cytosolic staining with detectable stronger signal intensity around the nuclear area seen using the DNA stain DAPI and the nuclear marker TgENO2 (Fig. S3 D). Analysis of signal intensity provided validation that some of the TgTom40^83–155^ signal colocalizes with the nuclear marker TgENO2 (Fig. S3 D). Furthermore, the expression of TgTom40^83–155^ in a line with Myc-tagged TgNup302 also resulted in the observation of overlap between the signals (Fig. S3 E). As a control, we measured the signal overlap of the cytosolic marker TgCDPK1 with Myc-tagged TgNup302 and showed that the pattern of TgCDPK1 signal intensity does not coincide with that of TgNup302; however, there are areas of overlap, as expected since TgCDPK1 is found all around the cell. Together these observations provide indirect evidence that TgTom40^83–155^ might be recruited to the nucleus which is in support of the possibility that it may interact with components of the nuclear pore.

**Figure 3.**
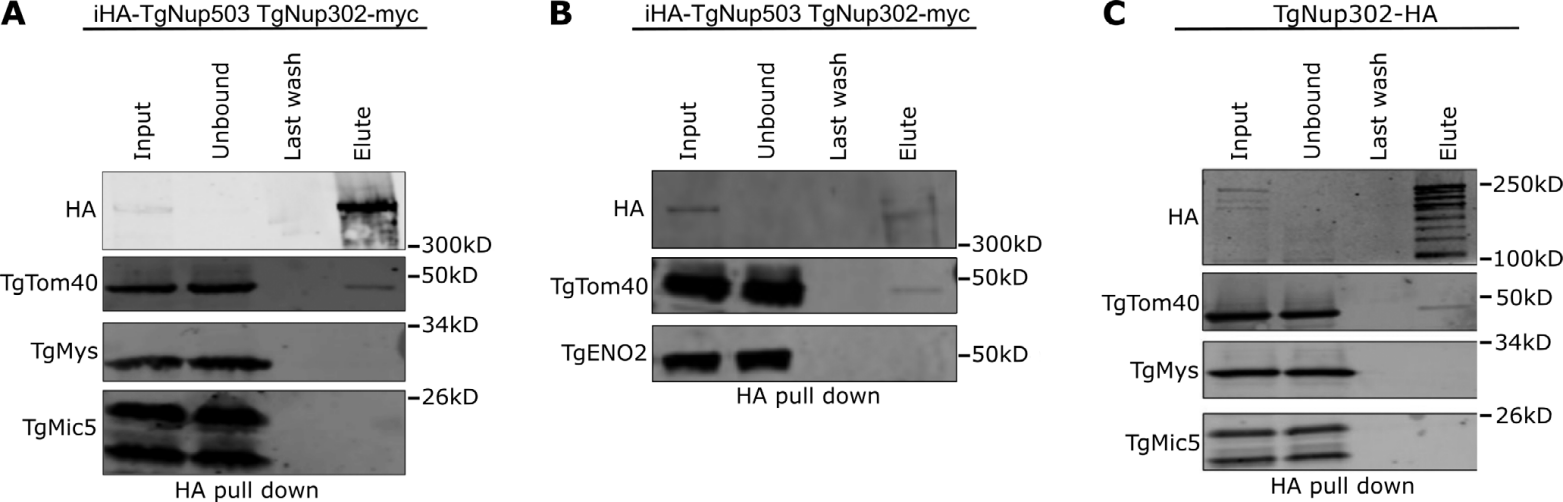
**Validation of the interaction of TgNup503 and TgNup302 with TgTom40. (A and B)** The interaction between HA-TgNup503 and TgTom40 was examined via anti-HA pull-down (using iHA-TgNup503HA TgNup302-myc line) followed by Western blotting probing for HA and TgTom40, as well as TgMys (unrelated protein in outer mitochondrial membrane) and TgMic5 (unrelated parasite protein) as negative controls (A) and for HA and TgTom40, as well as TgENO2 (unrelated nuclear protein) as negative control (B). **(C)** Interaction between TgNup302-HA and TgTom40 was probed as well by anti-HA pull-down (using TgNup302-HA line) followed by Western blotting probing for the same antibodies as used in A. Source data are available for this figure: SourceData F3.

As an additional way to validate the interaction between the nuclear pore and the TOM translocon, we repeated the pull-down of HA-tagged TgNup503, this time subjecting the fractions to Western blot analysis with anti-TgTom40 antibodies (Fig. 3, A and B). TgTom40 was detected in the pull-down fraction (Fig. 3, A and B), whereby the absence of other mitochondrial (TgMys), nuclear (TgENO2), and apical organellar (the microneme protein TgMic5) markers from the same fraction provides support for the specificity of the observed interaction (Fig. 3, A and B). The signal of TgTom40 in these experiments suggests that only a small fraction of the copies of this protein in the cell is involved in the interaction. This is in line with the expectation that most of the TgTom40 molecules in the cell are likely not engaged in mediating this contact as it primarily plays a role in protein translocation into the mitochondrion. Moreover, since the observed nuclear-mitochondrial contacts occurred in 46.3% of mitochondria profiles in sections that had both organelles in them, and that the nuclear pore was only seen in ∼15% of cases, it is likely that this putative nuclear pore-mediated contact is rare, and thus the number of tether molecules required is likely small.

Since TgNup503 localization seems to be spread beyond the nucleus in immunofluorescence, there is a risk that the interaction with TgTom40 observed by Western blot may occur away from the nuclear envelope in other parts of the ER. We wanted to examine whether TgNup302, which is tightly localized around the nuclear genome, is also involved in the interaction with TgTom40. Previous pull-down of TgNup302 did not recover TgTom40 (Courjol et al., 2017); however, the conditions were different: the previous experiment was performed from a combined nuclear extract and insoluble cellular fractions, and followed by mass spectrometry, while here we used the full cell lysate for the pull-down before analysis via Western blot. We thus performed the TgNup302 pull-down using the same conditions used for TgNup503 and analyzed the fractions by Western blot. Pull-down of HA-tagged TgNup302 resulted in the enrichment of multiple forms of TgNup302, in line with its reported processing (Courjol et al., 2017). Again, Western blot analysis showed that TgTom40 is found in the elution, but not the unrelated controls (Fig. 3 C), supporting the specificity of the observed interaction. Taken together, these observations demonstrate an interaction between components of the nuclear pore and the TOM translocon, in line with a potential role for those complexes as tether components for the newly identified contact.

### Depletion of TgNup503 results in mitochondrial morphological defects and reduced contacts between mitochondria and nucleus

**Figure S4.**
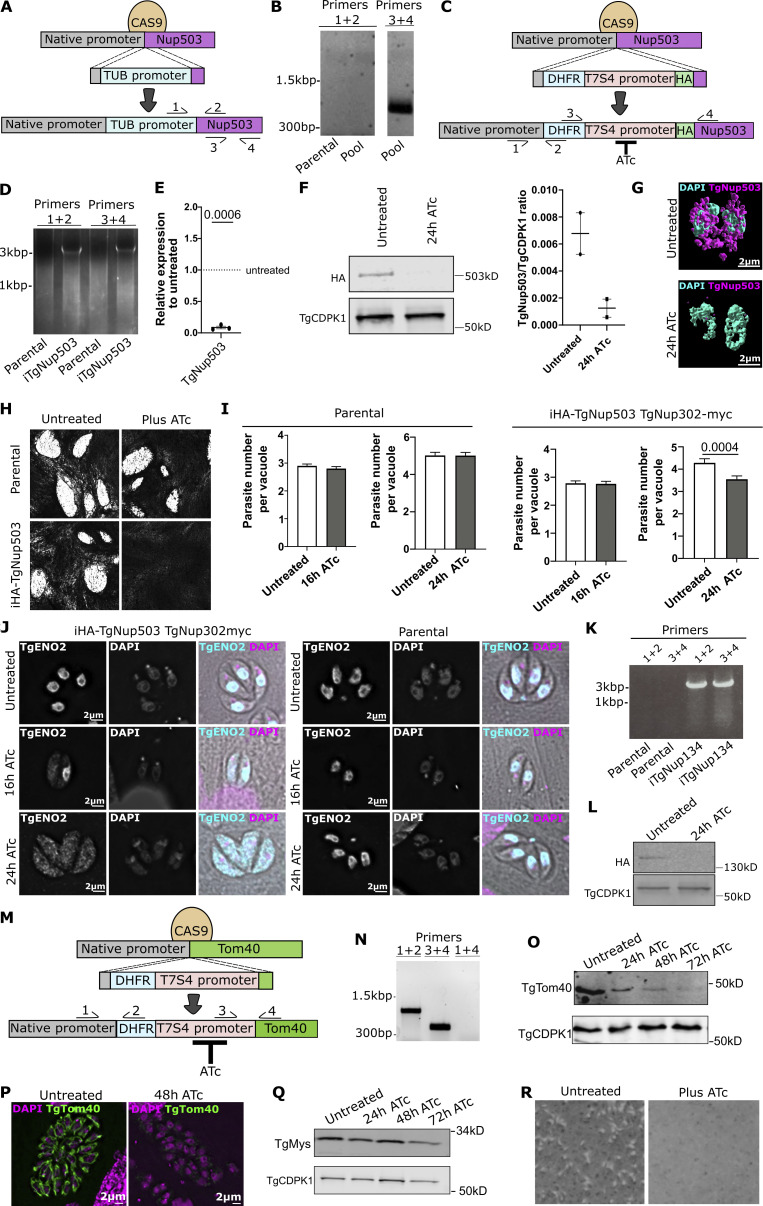
**Genetic manipulations of TgNup503, attempts for overexpression and generation, and analysis of inducible knock-down. (A)** A scheme of the genetic manipulation designed to generate a TgNup503 overexpression line, where, using CRISPR/CAS9, a new promoter (the strong *T. gondii* tubulin promoter, TUB light blue) would be inserted displacing the native TgNup503 promoter (grey). The scheme also shows the location of the primers used to test for integration. **(B)** One example of the four genetic manipulation attempts shows a lack of promoter integration (primers 1 + 2), while primers 3 + 4 confirm successful gDNA extraction as a positive control. **(C)** Scheme showing the genetic manipulation to generate the inducible depletion where, using CRISPR/CAS9, the native promoter is displaced and the gene of interest (*TgNup503*) is now under the control of the repressible promoter (T7S4) and contains an HA tag. Gene expression can be shut down by the addition of anhydrous tetracycline (ATc). The position of the primers used in D are indicated. **(D)** PCR validation of the promoter integration to generate inducible TgNup503 (i-HA-TgNup503). **(E)** qRT-PCR showing the downregulation of *TgNup503* expression 24 h post ATc addition (this experiment was performed with the parasite line resulting from the manipulation in D iHA-TgNup503 TgNup302-myc). Error bars display mean with SEM, data were analyzed with one sample *T* test (three biological replicates). **(F)** Western blot showing TgNup503 loss 24 h post ATc addition, TgCDPK1 was used as a loading control. Staining was done with secondary fluorescent antibodies followed by signal quantification. Error bars show mean with SEM, data were analyzed with two-sided unpaired *T* test (two biological replicates) (P = 0.0811). **(G)** SR image showing loss of TgNup503 24 h post ATc addition. **(H)** Plaque assays showing lack of growth of iTgNup503 upon TgNup503 depletion via ATc treatment. As a control, the growth of the parental line was unaffected by the presence of ATc. **(I)** Division assay showing growth of parental and iHA-TgNup503 TgNup302-myc line without ATc and at 16 and 24 h post addition of ATc addition. Three biological replicates, total of 300 vacuoles counted. Error bars display mean with SEM, data were analyzed with unpaired Wilcoxon test. **(J)** TgENO2 nuclear import assay in iHA-TgNup503 TgNup302-myc line and parental line in untreated cells and 16 and 24 h post addition of ATc. Scale bar is 2 μm. **(K)** PCR validation of the promoter integration to generate inducible TgNup134 (with the same strategy as shown in the scheme in C). **(L)** Western blot showing TgNup134 loss 24 h post ATc addition. TgCDPK1 was used as a loading control. **(M)** Scheme showing the genetic manipulation to generate the inducible depletion where, using CRISPR/CAS9, the native promoter is displaced, and the gene of interest (*TgTom40*) is now under the control of the repressible promoter (T7S4). Gene expression can be shut down by addition of anhydrous tetracycline (ATc). The position of the primers used in B are indicated. **(N)** PCR validation of the promoter integration to generate inducible TgTom40 (iTgTom40). **(O)** Western blot showing TgTom40 loss at 48 h post ATc addition, TgCDPK1 was used as a loading control. **(P)** Immunofluorescence images showing loss of TgTom40 at 48 h post ATc addition. **(Q)** Western blot showing that the mitochondrial marker TgMys remains at the same level at the 48 h time point when TgTom40 is almost fully lost. TgCDPK1 was used as a loading control. **(R)** Plaque assays showing lack growth of iTgTgTom40 upon TgTom40 depletion via ATc treatment. Source data are available for this figure: SourceData FS4.

We reasoned that if the nuclear pore mediates the observed contacts, then overexpression of its components would result in higher contact abundance. However, four independent attempts to replace the promoter of TgNup503 with the stronger constitutive *T. gondii* tubulin promoter failed (Fig. S4, A and B). On the other hand, a replacement with a weaker promoter was possible (Fig. S4, C and D), suggesting that overexpression of TgNup503 is not tolerated in the cells. As an alternative strategy, we attempted the opposite approach: we tested the effect of TgNup503 depletion on the corresponding contacts. We generated an inducible knock-down of TgNup503 via our promoter replacement strategy (Sheiner et al., 2011) whereby treatment with anhydrotetracycline (ATc) results in downregulation of the gene of interest (Fig. S4, C–E). We included an HA tag upstream of the new promoter to follow the protein level (Fig. S4 C). Using the tag, we confirmed the depletion of TgNup503 upon treatment with ATc by Western blot and immunofluorescence (Fig. S4, F and G). As expected for a nuclear pore protein, depletion of TgNup503 results in a growth defect as seen by the inability of the mutant *Toxoplasma* to plaque the monolayer of the host cells within which they grow, as well as by a delay in intracellular replication (Fig. S4, H and I). Replication analysis at 16 and 24 h enabled us to pinpoint the onset of growth defect at 24 h (Fig. S4 I). Finally, as demonstrated via transient gene disruption in the previous study of the nuclear pore in *T. gondii* (Courjol et al., 2017), stable inducible depletion of TgNup503 results in a redistribution of the nuclear proteins TgENO2 from the nucleus to the cytosol, indicating a defect in nuclear-cytoplasmic shuttling (Fig. S4 J). This effect is not seen in the parental control and is partial at 16 h of ATc treatment and nearly complete at 24 h of TgNup503 depletion (Fig. S4 J). Together these observations validate the depletion of TgNup503 in our system and highlight 24 h as the time point for observing both a full defect of the nuclear pore function and a defect in parasite replication.

**Figure 4.**
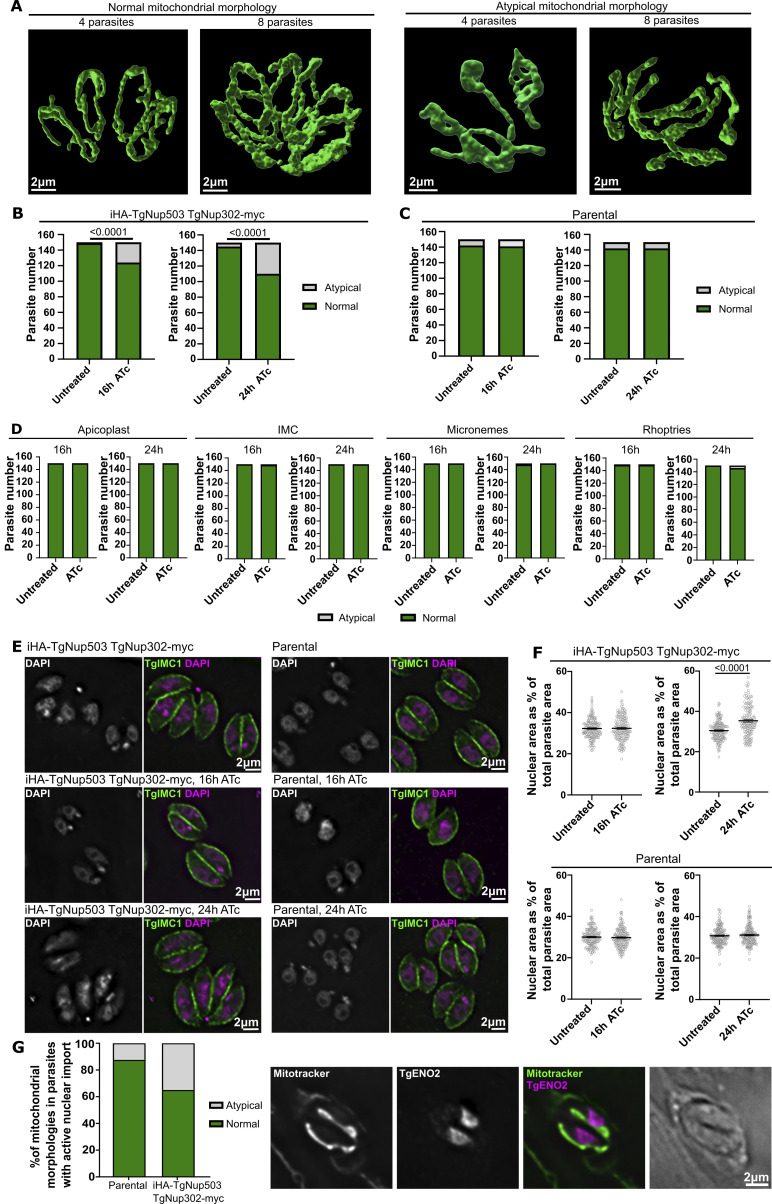
**TgNup503 depletion results in a specific mitochondrial morphology defect. (A)** Representative SR microscopy images of mitochondria (stained with TgTom40 antibody, green) of wild type (normal) mitochondrial morphology and mitochondrial morphological defects with more compact, “stringy” shape (atypical). Left image of each morphology shows the mitochondria of four parasites and the right images show eight. **(B)** Quantification of mitochondrial morphology (using confocal microscopy images, where the mitochondria were immunostained with anti-TgTom40) in iHA-TgNup503 TgNup302-myc line upon TgNup503 depletion (+ATc) for 16 and 24 h (three biological replicates per time point, total of 150 parasites). Data were analyzed with two-sided Fisher’s exact test. **(C)** Quantification of mitochondrial morphology in parental line treated with ATc for 16 and 24 h as control (three biological replicates per time point, total of 150 parasites). Data were analyzed with two-sided Fisher’s exact test (NS). **(D)** Quantification of organelle morphology (apicoplast—anti-TgCPN60; IMC—anti-TgIMC1; microneme—anti-TgMic5; and rhoptries—anti-TgRop7) in iHA-TgNup503 TgNup302-myc line upon TgNup503 depletion (+ATc) for 16 and 24 h (three biological replicates per time point, total of 150 parasites). Data were analyzed with two-sided Fisher’s exact test (all NS). **(E)** Representative fluorescent microscopy images used for the quantification in F. Parasite lines iHA-TgNup503 TgNyp302-myc (top) and parental (bottom) were treated with ATc for 16 or 24 h and co-stained with anti-TgIMC1 (green) and the DNA dye DAPI (pink). **(F)** Quantification of nuclear area (marked by DAPI) relative to the total area of the parasite (marked by IMC1) in iHA-TgNup503 TgNup302-myc and parental lines (three biological replicates per time point, total of 150 parasites). Error bars display mean with SEM and data were analyzed with two-tailed unpaired *T* test. **(G)** iHA-TgNup503 TgNup302-myc and parental line were treated with ATc for 24 h and stained with TgENO2 to identify parasites where nuclear cytosolic shuttling is still functional and stained with mitotracker to visualize mitochondria and analyze their morphology. The graph shows the quantification of mitochondrial morphologies (two biological replicates, a total of 40 parasites, statistical analysis not carried out).

In previous studies of mitochondrial membrane contact sites, including work in *Toxoplasma* (Mallo et al., 2021; Oliveira Souza et al., 2022), disruption of a molecular tether component resulted in morphological changes of the organelles in contact. Analysis of mitochondrial morphology via super-resolution microscopy (Fig. 4 A) identified mitochondria with atypical morphology upon depletion of TgNup503 at 24 h after the addition of ATc. To quantify and define the timing of this defect’s onset, we analyzed fluorescent micrographs and found that the defect appears as early as 16 h after ATc addition (Fig. 4 B) and is seen in the mutant but not in the control parental line (Fig. 4 C). Analysis of four other cellular organelles: the endomembrane pellicles, named the inner membrane complex (IMC); the apical secretory organelles, micronemes, and rhoptries; and the plastid organelle named the apicoplast, all showed no significant morphological defect in these mutants (Fig. 4 D), demonstrating the specificity of the observed mitochondrial defect. Finally, analysis of the nuclear genome via imaging of parasites stained with the DNA dye DAPI detected enlarged nuclei in the mutant (Fig. 4 E), likely a result of the defect in nuclear pore functions. Importantly, the onset of the latter defect is observed at 24 h of ATc (Fig. 4 F), later than the onset of mitochondrial morphology defect supporting that the mitochondrial changes observed are likely not secondary to nuclear pore functional defects. To further characterize the sequence of events, we analyzed mitochondrial morphology in parental and TgNup503 lines treated with ATc for 24 h where nuclear-cytoplasmic shuttling defect is seen, while focusing on parasites still presenting nuclear staining of TgENO2, which indicates that shuttling is still active in those parasites (Fig. 4 G). We found that 35% of TgNup503 line parasites with active nuclear-cytoplasmic shuttling presented mitochondrial morphological defects (Fig. 4 G) in line with this defect being a direct rather than secondary outcome of TgNup503 depletion.

**Figure 5.**
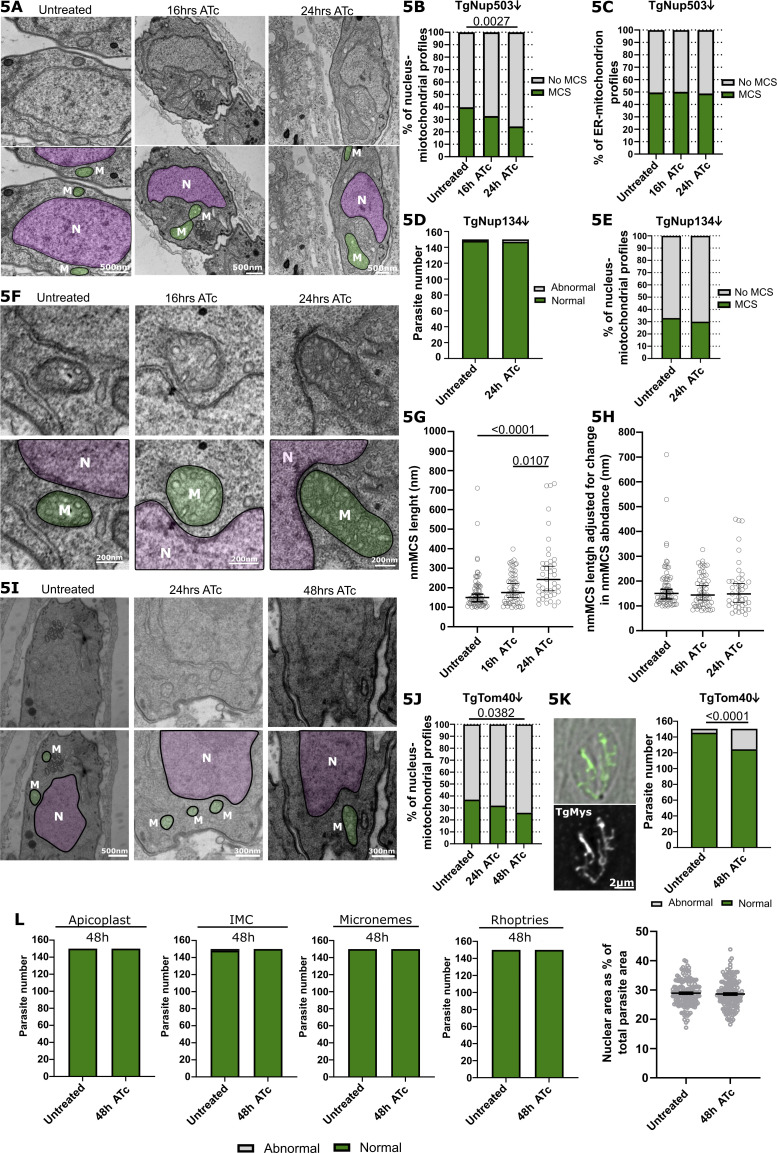
**Loss of nuclear-membrane contact sites upon TgNup503 or TgTom40 depletion. (A)** Representative EM images of untreated parasites and parasites treated with ATc for 16 and 24 h. Mitochondrion is highlighted in green and nucleus in pink. **(B)** Quantification of nuclear membrane contact sites abundance, defined, and analyzed by the same criteria as in Fig. 1, measured in EM images from parasites (iHA-TgNup503 line) untreated or treated with ATc for 16 and 24 h. Two biological replicates, 168–176 profiles analyzed per condition. Data were analyzed by χ-square test followed by two-sided Fisher’s exact test. **(C)** Quantification of ER-mitochondrion MCS from the same images used in B two biological replicates, 183–237 profiles analyzed per condition. Data analyzed by χ-square test (NS). **(D)** Quantification of mitochondrial morphology from the negative control iHA-TgNup134 line, untreated or treated with ATc for 24 h (three biological replicates per time point, total of 150 parasites). Data were analyzed with two-sided Fisher’s exact test (NS). **(E)** Quantification of nuclear membrane contact sites abundance, defined and analyzed by the same criteria as in Fig. 1, measured in EM images from parasites (iHA-TgNup134 line) untreated or treated with ATc for 24 h. Two biological replicates, 112–192 profiles analyzed per condition. Data were analyzed with two-sided Fisher’s exact test (all NS). **(F)** EM images illustrating the increased length of nmMCS upon TgNup503 depletion. Mitochondrion is highlighted in green and nucleus in pink. **(G)** Quantification of nmMCS length (iHA-TgNup503 line) upon TgNup503 depletion. Each measurement represents a nmMCS (two biological replicates per condition, 41–69 measurements per condition, outlier (1,523 nm) in untreated condition was removed from analysis). Error bars display a median with 95% CI. Data were analyzed with Kruskal–Wallis test. **(H)** Recalculated lengths of the nmMCS shown in F while applying the reduction in nmMCS abundance to each value. Error bars display the median with 95% CI. Data were analyzed with Kruskal–Wallis test (NS). **(I)** Representative EM images of untreated parasites and parasites, iTgTom40, treated with ATc for 16 and 24 h. Mitochondrion is highlighted in green and nucleus in pink. **(J)** Quantification of nuclear membrane contact sites’ abundance, defined and analyzed by the same criteria as in Fig. 1, measured in EM images from parasites (iTgTom40 line) untreated or treated with ATc for 24 and 48 h. Two biological replicates, 152–202 profiles analyzed per condition. Data analyzed by χ-square test followed by two-sided Fisher’s exact test. **(K)** Quantification of mitochondrial morphology from the iTgTom40 line, untreated or treated with ATc for 48 h (three biological replicates per time point, total of 150 parasites). Data were analyzed with two-sided Fisher’s exact test. **(L)** Quantification of organelle morphology (apicoplast—anti-TgCPN60; IMC—anti-TgIMC1; microneme—anti-TgMic5; and rhoptries—anti-TgRop7) in TgTom40 line upon TgTom40 depletion (+ATc) for 48 h (three biological replicates per time point, total of 150 parasites). Quantification of nuclear area (marked by DAPI) relative to the total area of the parasite (marked by IMC1) in iTgTom40 line (three biological replicates per time point, total of 150 parasites). Data were analyzed with two-sided Fisher’s exact test (all NS). For nuclear size, the error bars display mean with SEM, and data were analyzed with two-tailed unpaired *T* test.

We next examined EM images to analyze the abundance of the nuclear mitochondrial membrane contact sites following TgNup503 depletion (Fig. 5 A). We found a reduction in the observed contacts already at the 16-h time point, which grew more prominent at the 24-h time point (Fig. 5 B). As a control, we examined ER-mitochondrial membrane contact sites in those images. We saw no significant change in the abundance of ER-mitochondrial contacts at these time points (Fig. 5 C), suggesting that the observed effect on nuclear-mitochondrial contacts is specific. As an additional support for specificity, we generated another inducible depletion line, this time targeting TgNup134, a nuclear pore protein that was not found to interact with TgNup503 and TgNup302 (Table S1 and Courjol et al., 2017). We generated a promoter replacement line for TgNup134 and validated the integration and protein depletion via PCR and Western blot (Fig. S4, K and L). Analysis of mitochondrial morphology via immunofluorescence showed no change in morphology (Fig. 5 D). Analysis of EM sections revealed a mild trend of reduced nuclear-mitochondrial membrane contact sites in the untreated parasites compared with the iTgNup503 line; however, no change in the abundance of the nuclear mitochondrial membrane contact sites following TgNup134 depletion was seen (Fig. 5 E) in support of the specificity of the observed reduction upon TgNup503 depletion.

Our analysis of EM sections of the TgNup503-depleted parasites highlighted several cases of unusually long nuclear mitochondrial contacts compared with the wild type (Fig. 5 F), which might indicate that compensation is taking place. Analysis of the lengths of nuclear mitochondrial contacts in untreated parasites and at 16 and 24 h of ATc treatment revealed a trend of increased contact lengths upon TgNup503 depletion with significantly longer contacts at 24 h compared with untreated parasites (Fig. 5 G). We attempted to use the number of contacts seen and the lengths measured to evaluate the overall contact between the two organelles in the analyzed EM profiles of each condition. We thus applied the percentage drop of contact numbers to the measured contact lengths (for example, at 16 h of ATc treatment, contact abundance dropped by 17.9%, from 39.8% to 32.7%; hence we decreased each measured contact length by 17.9% for our calculation). Applying this calculation resulted in similar median lengths among the three conditions with no significant difference between untreated and TgNup503 depleted conditions (Fig. 5 H), in support of the prolonged contact reflecting a possible compensation response.

Taken together, the specific morphological changes and specific reduction of the number of nuclear mitochondrial contacts at an early time point of TgNup503 depletion, prior to defects of nuclear shuttling, enlarged nuclei, or replication, support a role of the nuclear pore in tethering the two organelles. Furthermore, the observation of elevation in contact length might point to a compensation mechanism in response to this reduction.

### Depletion of TgTom40 results in mitochondrial morphological defect and reduced contacts between mitochondria and nucleus, as observed upon TgNup503 depletion

The observed interaction between nuclear pore components and TgTom40 pointed to the latter as a likely tether partner. In the previous study describing the TOM70-Cnm1 tether, the deletion of one tether component (Tom70) affected the signal distribution of the other (Cnm1) (Eisenberg-Bord et al., 2021). We reasoned that likewise, the depletion of TgNup503 might be accompanied by a redistribution of the TgTom40 signal. We showed that while the localization of the nuclear pore marker TgNup302 remains tight around the nucleus (Fig. S1 J) and shows no change in its overall signal volume upon TgNup503 depletion at the 24 h time point (Fig. S1, K and L), the overlap of TgTom40 signal onto the TgNup302 signal is enhanced upon the depletion (Fig. S1 M). This was not a result of elevated expression of TgTom40 as seen in its unchanged overall signal volume (Fig. S1 M) and via Western blot (Fig. S1 N). The unrelated outer mitochondrial membrane marker TgMys remains unchanged in expression and signal volume and shows a mild and not significant change in the signal overlap with TgNup302 (Fig. S1, L–N), which provides support for a potentially specific change in TgTom40 signal distribution. These observations could be an outcome of TgTom40 molecules that no longer concentrate at the contact but instead contribute to signal overlap in other places where the mitochondrion and nucleus are in less tight proximity, which is picked up by the low resolution of the method. However, it cannot be excluded that the enhanced overlap is simply an outcome of the enlarged nuclei seen at the same time point. The trend of elevated signal overlap with TgMys, albeit not significant, supports the latter scenario.

Thus, to provide support for the role of TgTom40 in this new contact via a different method, we tested the effect of TgTom40 depletion on the nuclear mitochondrial contacts, as done above for TgNup503. We generated an inducible knock-down of TgTom40 via the same promoter replacement strategy (Fig. S4, M and N). Using anti-TgTom40 antibodies, we confirmed its depletion after 48 h of ATc treatment by Western blot and IFA (Fig. S4, O and P). Western blot of the unrelated mitochondrial marker TgMys showed that it remains present until 72 h of TgTom40 depletion (Fig. S4 Q) providing control for the specificity of the observed TgTom40 reduction. As expected for an essential component of the mitochondrial protein import translocon, depletion of TgTom40 results in a growth defect observed by a plaque assay (Fig. S4 R). Together these observations validate the depletion of TgTom40 in our system and highlight 48 h as the time point of observing near-complete protein depletion.

We next examined the EM section of parasites with depleted TgTom40 for 24 and 48 h to analyze the abundance of the nuclear mitochondrial membrane contact sites following this depletion (Fig. 5 I). We found a reduction in the observed contacts already at the 24-h time point, which grew more prominent at the 48-h time point (Fig. 5 J), in line with the timing of TgTom40 protein level reduction. As in the case of TgNup503 depletion, the reduction in contacts was accompanied by mitochondrial morphology changes seen by immunofluorescence (Fig. 5 K), while analysis of the morphology of other organelles at 48 h of depletion shows no significant change in the morphology of the IMC, rhoptries, and apicoplast or in the morphology or size of the nuclei (Fig. 5 L).

Taken together, the specific morphological changes and reduction of nuclear mitochondrial contacts upon TgTom40 depletion support the role of the mitochondrial TOM translocon in tethering the two organelles, while the possibility that the reduction seen might be an outcome of an overall mitochondrial biogenesis defect upon TgTom40 depletion cannot be excluded.

### Depletion of TgNup503 results in a specific yet mild enhancement of mitochondrial membrane potential

**Figure 6.**
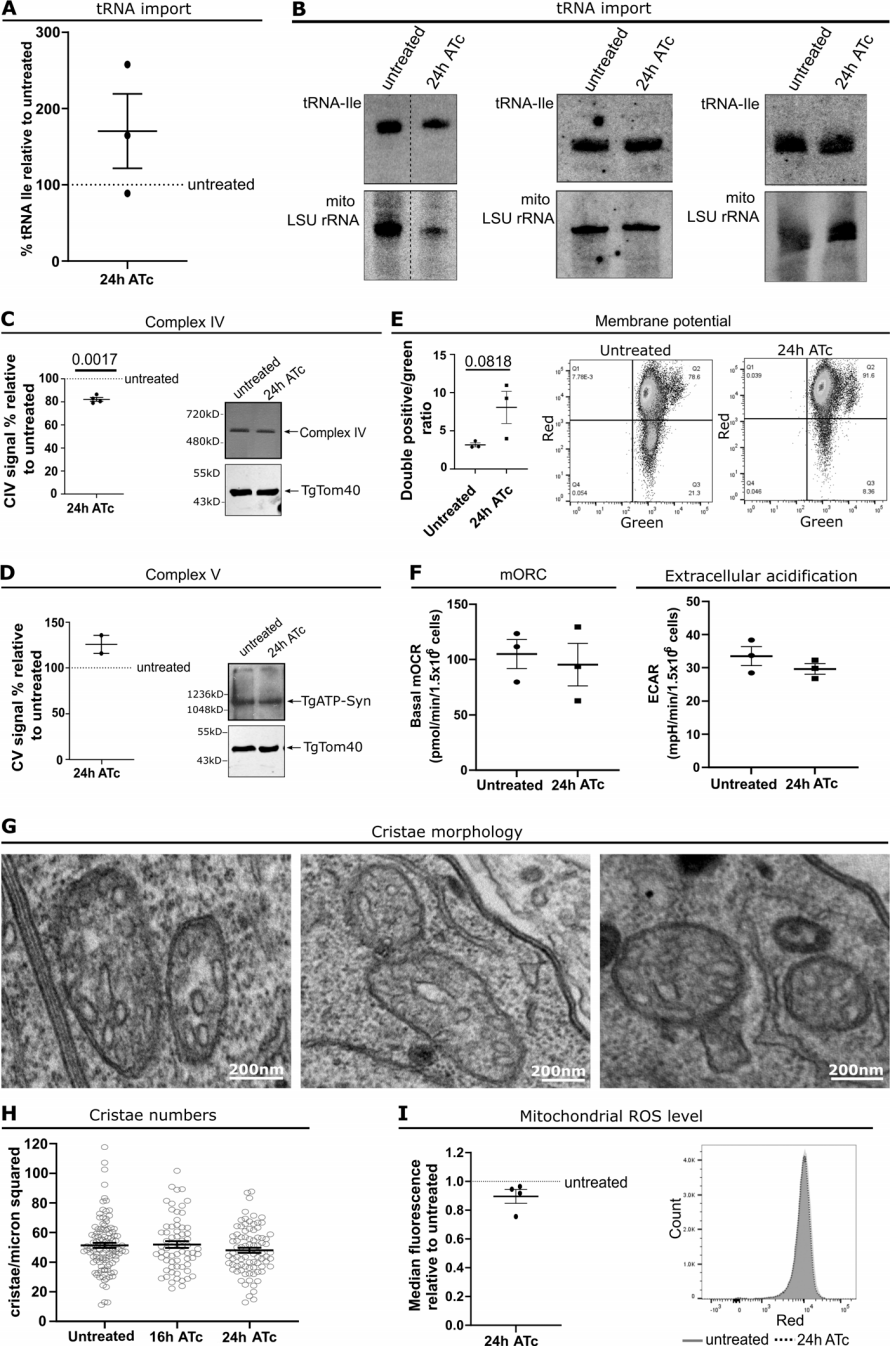
**TgNup503 depletion results in a trend of mild enhanced mitochondrial potential and have no effect on other functions of the respiratory chain. (A)** Quantification of mitochondrial tRNA levels in treated (24 h) relative to untreated parasites (three biological replicates all shown in I). Error bars show mean with SEM. Data were analyzed with one sample *T* test compared with untreated (NS). **(B)** Individual Northern blots were used for quantification in H. Top panels show Northern blots with probes against tRNA^Ile^ whereas the bottom panels show Northern blots with probes against a mitochondrial rRNA for loading control of mitochondrial fraction. **(C)** A representative image and graph of measurements from four biological replicates of in-gel assembly and activity assay for complex IV. Error bars display mean with SEM. Data were analyzed with one sample *T* test comparing to untreated. **(D)** Blue native page followed by Western blot to assess assembly of complex V (representative image of biological replicates). Error bars display mean with SEM. Two biological replicates, data were analyzed with one sample *T* test comparing to untreated (NS). **(E)** Membrane potential was assessed using JC-1 dye where membrane potential results in movement of the dye into the mitochondrion where it aggregates changing from green to red. Error bars show mean with SEM, three biological replicates, data were analyzed with two-tailed unpaired *T* test (NS). **(F)** Basal mitochondrial oxygen consumption rates (basal mOCR) and extracellular acidification rate (ECAR) of iHA-TgNup503 TgNup302-myc grown in the presence or absence of ATc for 24 h, three biological replicates. Error bars display mean with SEM. Data were analyzed with two-tailed unpaired *T* test (NS). **(G)** Representative images of mitochondria from 24 h ATc treated iHA-TgNup503 TgNup302-myc parasites showing their cristae. **(H)** Cristae count from EM of iHA-TgNup503 TgNup302-myc, untreated, and ATc treated for 16 and 24 h. Two biological replicates, 65–111 mitochondria analyzed per condition. Error bars display mean with SEM. Data were analyzed with one-way ANOVA (NS). **(I)** Level of mitochondrial ROS was assessed with MitoSOX dye and flow cytometry. Error bars show mean with SEM, four biological replicates. Data were analyzed with one sample *T* test compared with untreated (NS). Source data are available for this figure: SourceData F6.

We considered the possible functions of the new contact. The *Toxoplasma* mitochondrial genome encodes no tRNAs and thus tRNAs are imported to support translation, likely in their amino-acylated state (Esseiva et al., 2004; Pino et al., 2010). In *Trypanosomes*, another group of protozoan parasites where all mitochondrial tRNAs are nuclear-encoded, the import takes place via the mitochondrial protein translocon (Niemann et al., 2017; Shikha et al., 2020). Seeing that aminoacylation of tRNAs can take place in the nucleus which is important for tRNA export (Lund and Dahlberg, 1998; Sarkar et al., 1999), we considered whether the observed contact between the nuclear pore and the mitochondrial protein translocon might mediate mitochondrial tRNA import in *Toxoplasma*. To identify proteins involved in mitochondrial tRNA import, we performed pull-down with biotinylated tRNA, as done previously in *Trypanosomes* (Seidman et al., 2012). We selected tRNA^Ile^ that was experimentally shown to be mitochondrially imported in *Toxoplasma* (Pino et al., 2010) as “bait” and used tRNA^Met-i^ which is not imported into mitochondria (Pino et al., 2010) as a negative control, and pull-downs were performed five times with each tRNA (Table S2). Seven proteins were pulled down with tRNA^Ile^ at least twice and never with tRNA^Met-i^ across the five repeats (Table 2). Interestingly, TgNup302 was found among those seven, providing support to possible involvement of the nuclear pore in mitochondrial tRNA import. We thus examined whether our depletion of TgNup503 might affect mitochondrial tRNA import through Northern blot analysis of steady-state levels of tRNAs in a digitonin-extracted organelle fraction as described previously (Esseiva et al., 2004; Pino et al., 2010). Following three repeats, we observed no significant or consistent reduction in mitochondrially imported tRNA after 24 h of ATc treatment (Fig. 6 A), albeit finding high variability among the different experiments (Fig. 6 B). Thus, a direct role for this contact in mitochondrial tRNA import was not supported.

**Table 2.** Interactions of tRNA identified via pull down

Gene id (ToxoDB)	Predicted function	Times found in pull-down		Predicted localization
		tRNA^Ile^	tRNA^Met-i^	
TGME49_219470	Hypothetical protein	2	0	Mitochondrion^a^^,^^b^
TGME49_220950	Hypothetical protein (MAF1b)	2	0	Elsewhere^a^^,^^b^
TGME49_246540	Cytochrome c1, heme protein	2	0	Elsewhere^a^^,^^b^
TGME49_253740	Hypothetical protein	2	0	Nucleus^a^^,^^b^
TGME49_259640	Nucleoporin autopeptidase (Nup302)	2	0	Nucleus^a^^,^^b^
TGME49_267660	Hypothetical protein	2	0	Nucleus^a^^,^^b^
TGME49_297780	DNA gyrase B	2	0	Elsewhere^a^^,^^b^

a Mitochondrial prediction tools: Predotar, Mitoprot, Mitofates, TargetP-2.0, iPSORT.

b Nuclear prediction tool: cNLS Mapper.

Mitochondrial respiration and energy conversion are essential to the survival of most eukaryotes including *Toxoplasma* (Huet et al., 2018; Seidi et al., 2018; Hayward et al., 2021; Maclean et al., 2021). Coordination between the nucleus and the mitochondrion is essential to control respiration and different pathways of mitochondria-nuclear signaling are proposed to mediate this critical coordination (Eisenberg-Bord and Schuldiner 2017). To examine if the observed contact might contribute to this communication, we analyzed the mitochondrial respiratory chain and ATP synthase (complex V) in the TgNup503 depleted parasites. First, we examined the formation of complexes IV and V for which we have established assays in *Toxoplasma* (Lacombe et al., 2019; Maclean et al., 2021). Native PAGE migration and in-gel activity assay for complex IV, or Western blot for complex V, suggested no change in complex formation upon TgNup503 depletion (Fig. 6, C and D). Furthermore, measuring complex IV in-gel cytochrome c oxidation activity suggested only a minor reduction in this activity upon TgNup503 depletion (Fig. 6 C). Likewise, a complex II in-lysate activity assay also showed no significant difference upon TgNup503 depletion (data not shown). Next, we measured the mitochondrial membrane potential, which results from the proton pumping activity of complex III and IV that is coupled to their electron transport activity. Using the dye JC-1 as previously described (Maclean et al., 2021), we detected a trend of enhanced mitochondrial membrane potential upon TgNup503 depletion at 24 h of incubation with ATc, albeit not significant compared with untreated parasites (Fig. 6 E). Enhanced membrane potential is sometimes linked to an overall enhanced respiration and we thus examined mitochondrial respiration using several methods. Examination of both the basal mitochondrial oxygen consumption rate and an extracellular acidification rate via a seahorse assay (Hayward et al., 2022) showed no change in both activities upon TgNup503 depletion (Fig. 6 F). Further, we analyzed mitochondrial cristae using EM since changes in respiration are often accompanied by a corresponding change in mitochondrial cristae structure. Our analysis showed that the number of cristae per square micron of mitochondrion remains unchanged; however, we could not exclude a subtle change in cristae shape (Fig. 6, G and H). Finally, the superoxide radical (O2^−^) is a byproduct of the respiratory chain, which results from electron leakage. Enhanced mitochondrial electron transport thus often results in elevated reactive oxygen species (ROS). We analyzed the mitochondrial ROS levels at 24 h, the time points where a trend of enhanced membrane potential was observed but found no changes in mitochondrial ROS under these conditions (Fig. 6 I). Together these observations suggest that TgNup503 depletion leads to a slightly elevated proton concentration in the mitochondrial intermembrane space and that this change is independent of respiration activity for which all our direct and indirect analyses show no change upon TgNup503 depletion.

## Discussion

The evolution of pathways of communication between the nucleus and mitochondria was a prerequisite for mitochondrial acquisition in the eukaryotic ancestor. Accordingly, many nuclear-mitochondrial communication pathways enable the coordination between these organelles, and the role of membrane contact sites in mediating and regulating some of these pathways is just starting to emerge (Ovciarikova et al., 2022b).

So far only two nuclear-mitochondrial contacts have been assigned as molecular machinery, one in yeast and the other in human cells, where tethering is mediated by nuclear envelope or matrix proteins, respectively (Desai et al., 2020; Eisenberg-Bord et al., 2021). Here, through studying the protozoan *T. gondii*, we report a new nuclear-mitochondrial contact site that is mediated by a previously uncharacterized interaction between the nuclear pore and the mitochondrial protein translocation complexes. We provide evidence for an interaction that involved components of both complexes, TgNup503, TgNup302, and TgTom40, and we demonstrate that the depletion of TgNup503 or of TgTom40 results specifically in reduced contacts between the two organelles, which is linked to specific defect in mitochondrial morphology, and, in the case of TgNup503, to a mild enhancement of the mitochondrial membrane potential.

Why would the nuclear pore be involved in mediating contact with the mitochondrion? Over the past two decades of exploring the molecular machinery of membrane contact sites, a theme was formed in the literature whereby many of the tethers involved components that are ancient in eukaryotic history and that are abundant in the membranes in contact. This holds true for the involvement of the ubiquitous and abundant outer mitochondrial membrane protein porin/VDAC in several mitochondrial contacts (Liu et al., 2019; Mallo et al., 2021). Another example is one of the first membrane contact site tethers described, the ER-mitochondria encounter structure (ERMES) (Kornmann et al., 2009), which was found to be archaic (Wideman et al., 2013), despite its absence in some eukaryotic clades. We postulate that when contact sites evolved, proteins or structures that were abundant in the membranes were repurposed as tethers and this happened with the nuclear pore. The involvement of Tom40 is also in line with this hypothesis as it is an ancient protein (Fukasawa et al., 2017). In fact, components of the mitochondrial outer membrane protein translocation complex, TOM, play a role in a number of mitochondrial contacts with different organelles and in divergent organisms, including the ER-mitochondrial contact (Lahiri et al., 2014; Namba, 2019), mitochondrial-vacuole contact (Elbaz-Alon et al., 2015; Murley et al., 2015; González Montoro et al., 2018), and now the nuclear-mitochondrial contacts reported previously (Eisenberg-Bord et al., 2021) and here.

The discovery of a tether that involves the nuclear pore, the site of tRNAs export from the nucleus, and the mitochondrial protein translocon, which mediates mitochondrial tRNA import (Niemann et al., 2017; Shikha et al., 2020), tempted us to explore the possibility that this contact may be involved in mitochondrial tRNA import. In other organisms where mitochondrial tRNA import was studied in detail, a recurring theme is that a protein with dual function serves as a “recruiter” that separates the tRNAs destined for the mitochondrion from the greater cytosolic pool and directs them to the mitochondrion. For example, in yeast, the mitochondrially imported tRNA^Lys^ is specifically recognized by an isoform of the glycolytic enzyme enolase (Entelis et al., 2006). Likewise, in *Trypanosomes*, the specificity of tRNAs imported to the mitochondrion is determined through binding to the translation elongation factor 1a (Bouzaidi-Tiali et al., 2007). In our hypothetical scenario, we speculated that a component of the nuclear pore, e.g., TgNup302, may serve as the recruiter and that the proximity to the TOM translocon mediated by the contact may facilitate the movement of tRNA molecules into the mitochondrion. Our mitochondrial tRNA import assays did not support this proposal; however, two caveats should be highlighted: first, our assays detect the steady state level of organellar tRNA rather than analyzing only tRNA imported after TgNup503 depletion; second, we detected high variability between experiments. Thus, while our data provide no support for it, we cannot fully exclude the possibility of this new contact being involved in mitochondrial tRNA import with the tools available to us.

Depletion of TgNup503 results in a possible minor enhancement of the mitochondrial membrane potential, while the other mitochondrial functions that we could measure remain unchanged. While we cannot exclude that the aberrant mitochondrial shape in itself led to the change seen in the membrane potential, it could also be that the contact described here mediates a form of nuclear-mitochondrial communication necessary to control mitochondrial membrane potential. Enhanced membrane potential has two main routes, an enhanced activity of the mitochondrial electron transport chain, which results in higher proton concertation in the intermembrane space, and an inhibition of the ATP synthase activity of the mitochondrial complex V, which results in reduced harvest of those protons. Our findings of no change in respiratory complex formation, mitochondrial oxygen consumption, cristae number, and ROS levels suggest against enhanced electron transport and coupled proton pumping activities in our conditions. This leaves the possibility of ATP synthase inhibition more likely. Interestingly, the mitochondrial ATP synthase inhibitory factor 1 was shown to trigger a ROS-mediated retrograde prosurvival response in human carcinoma cells; however, the mechanism controlling this trigger is unknown (Formentini et al., 2012). Whether the herein-described nucleus–mitochondria contact has a role in regulating ATP synthase activity and inhibition remains to be studied. Having the tether molecules at hand should help investigate this possibility or explore other functions.

The two nuclear-mitochondrial tethers studied previously involve proteins that are residents of the nuclear envelope or matrix rather than the nuclear pore. Does this difference reflect a divergence between lineages or the existence of different types of tethers between the two organelles? Our data show that depletion of either TgNup503 or TgTom40 leads to a partial reduction in the observed mitochondrial-nuclear contacts rather than complete ablation, as has been seen in other contacts previously (ER-PM [Manford et al., 2012]; ER-endosome [Henne et al., 2015]; and ER-mitochondria [Simmen et al., 2005]) and attributed to multiple different tethers between the same two organelles. Not only that, the reduction of nuclear-mitochondrial contacts upon TgNup503 depletion was accompanied by a growth in the length of the contacts that remained (Fig. 5, F–H), highlighting a possible compensation response potentially mediated by another tether. These observations support the presence of another tether that may mediate another type of nuclear-mitochondrial contacts in *Toxoplasma*. For example, while a homolog of Cnm1 is not readily found in the *Toxoplasma* genome, it is still possible that another nuclear envelop protein is involved in forming a contact that mediates the proposed transfer of phosphatidylcholine from the nuclear envelope to the mitochondrion. Further support for the possibility of multiple tethers for nuclear-mitochondrial contacts in eukaryotes is provided by the identification of additional effectors of the nucleus–mitochondria contact on top of Cnm1 in the screens performed by Eisenberg-Bord et al. (2021), and this may well be the case for *Toxoplasma* too. Finally, multiple types of contacts, linked by different tethers and mediating different functions, have been identified for the ER and mitochondria (Herrera-Cruz and Simmen 2017), providing precedence for this scenario. Thus, we are likely just at the beginning of the journey of revealing the array of nuclear-mitochondrial contacts in eukaryotes, and our work may pave the way to the identification of nuclear pore-mediated contacts in other organisms.

## Materials and methods

### Electron microscopy

Samples for TEM were fixed in 2.5% glutaraldehyde in 0.1 M cacodylate buffer and further postfixed in 1% OsO_4_ + 1.25% potassium ferrocyanide + 5 mM CaCl_2_ in 0.1 M cacodylate buffer, followed by en bloc staining using 1.0% aqueous uranyl acetate RT in the dark. Samples were dehydrated in ascending acetone series and embedded in epoxy resin. Ultrathin sections (60–70 nm) were obtained via a Leica UC6 ultramicrotome, and collected grids were imaged using a Fei Tecnai Spirit 120 kV transmission electron microscope.

### Electron tomography

For 3D electron tomography, 250 nm–thick sections of free tachyzoites processed for TEM (as described previously) were collected onto formvar-coated 100-mesh copper grids. Colloidal gold (10 nm in size) was added onto grids prior to imaging. Images were recorded in tilt series covering ±60° at 1° increment intervals in a Jeol 1400 transmission electron microscope (Jeol) operating at 120 kV equipped with an AMT UltraVue camera. Tilt series were aligned by cross-correlation and tomogram reconstruction calculated by weighted back projection using Etomo from IMOD software package. Segmentation and generation of the 3D model were performed using the 3dmod program of the same software package.

### Immunofluorescent assay

Parasites were cultured in human foreskin fibroblasts seeded on a glass coverslip. For mitotracker treatment, the cells were treated with a 50-nm mitotracker for 30 min at 37°C followed by 4x DMEM and 1x PBS wash prior to fixation. At a specified time point, the cells were fixed with 4% PFA for 20 min at RT, blocked, and permeabilized in PBS/0.2% Triton-X100/2% bovine serum albumin (blocking buffer) for 20 min, RT followed by incubation with primary antibodies for 1 h at RT: myc (1:1,000; Cell Signalling), HA (1:1,000; Sigma-Aldrich), TgTom40 (1:1,000; gift from Giel van Dooren, Australian National University, Canberra, Australia), TgMys ([Ovciarikova et al., 2017], 1:1,000), TgCPN60 ([Agrawal et al., 2009], 1:1,000), IMC1 (1:1,000; gift from Gary Ward, University of Vermont, Burlington, VT), TgMic5 (1:1,000; gift from David Smith, Moredun Institute, Penicuik, UK), TgRop7 (1:1,000; gift from David Smith), Ty (1:800; gift from Giel van Dooren), Ty (1:1,000; Thermo Fisher Scientific), TgENO2 ([Courjol et al., 2017], 1:1,000), and CDPK1 (1:10,000; gift from Clare Harding, University of Glasgow, Glasgow, UK). Cells were washed three times with PBS/TritonX-100 and then incubated with secondary antibodies for 45 min at RT in the dark: AlexaFluor 594 or 488 (1:500; Invitrogen). Cells were washed three times with PBS/TritonX-100 and mounted on glass slides with DAPI Fluoromount-G (Southern Biotech).

For staining of extracellular parasites, glass coverslips were covered by poly-L-lysine solution (Sigma-Aldrich) for a minimum of 1 min at RT and then allowed to air-dry. Naturally lysed parasites were put onto the coverslips and spun gently (100–300 × *g* for 1–3 min) to allow the parasites to adhere to the coated coverslips. The cells were fixed and stained as described above.

### Imaging

Images were acquired with DeltaVision Core microscope (AppliedPrecision; GE Healthcare) with 0.2 μm increment, Photometrics CoolSNAP HQ2 CCD camera, 100× oil-immersion objective at RT using softWoRx Suite 2.0 acquisition software followed by image deconvolution (conservative with applied correction and medium filtering). Images were reconstructed and analyzed with FIJI ImageJ 64 software (Schindelin et al., 2015).

For super-resolution images, slides were imaged using Elyra PS.1 super-resolution microscope (Zeiss) with a 63×/1.4 NA oil-immersion objective, pco.edge (sCMOS) camera, and imaged at RT using ZEN black software (Zeiss) capturing the entire vacuoles depth in 0.091-μm increments. Images were acquired with a three-phase setting and structure illuminated and aligned in the imaging software. Images were reconstructed, and colocalization (Pearson’s coefficient in dataset volume) was measured with Imaris software (Oxford Instruments).

For the measurement of nuclear size, maximum projections were created in FIJI. The nuclear area (marked by DAPI) and parasite area (marked by TgIMC1) were traced manually and measured. Dividing parasites (daughter cells present) were excluded.

### Generation of transgenic *T. gondii* strains

The TgNup503 and TgNup134 iKD lines were generated using RH∆ku80TaTi (Sheiner et al., 2011) and a plasmid containing genomic fragments encompassing 2 kb upstream the gene and 2 kb from the predicted ATG to guide homologous recombination (TgNup503 promeoter F [5′-TTAATTAATCACGGTACCTGAAGTTCTTCATGTT-3′], TgNup503 promoter R [5′-ATGCATCCTTCTGCCGACGGAGCGAG-3′], TgNup503 gene F [5′-CCCGGGATGGCAAGCTCCGGTGGCGG-3′], TgNup503 gene R [5′-GCGGCCGCTGCGGACTCAGGACATGCGG-3′], TgNup134 promoter F ([5′-GGGCCCCCGTTCTCTCATTATCGGCGT-3′] TgNup134 promoter R [5′-CATATGATTTCCTTTCCCTGTCGGTGA-3′], TgNup134 gene F [5′-CCCGGGATGGATTGCCAGCCTCGGCA-3′] and TgNup134 gene R [5′-GCGGCCGCGCATATCCTCTCTGTCGGG-3′], TgNup134 3′ integration [5′-CTGGCCAGTCGAGGTCATGG-3′], TgNup134 5′ integration [5′-GTCGTTGTAGCAACCAGTGGT-3′], DHFR 3′ F [5′-CGTTCCCCATTGTGAACATC-3′], and DHFR 5′ R [5′-CTTCAGCTTGAGGCTTCTCC-3′]). To produce the Myc-tagged Nup302, and TGGT1_258970 and TGGT1_287500 proteins by the knock-in strategy, a DNA fragment of 2 kb upstream of the stop codon was amplified from genomic DNA and cloned in the pLIC-Myc-CAT plasmid as described in Courjol et al. (2017). These plasmids were transfected into the TgNup503 iKD strain and selection was performed using chloramphenicol. The TgNup302HA tagline was generated using the CRISPR/CAS9 system where a cut was introduced at the stop codon and an HA-CAT cassette flanked with 50 bp homology regions to upstream and downstream of the stop codon was inserted (TgNup302 gRNA F [5′-AAGTTCAGCGCTGCTCGAAGAACGCG-3′], TgNup302 gRNA R [5′-AAAACGCGTTCTTCGAGCAGCGCTGA-3′], TgNup302 cassette F [5′-CTGCATTCTGGATTGCGCTCAAGACAGCGCTGCTCGAAGAACGCAGGGAACCTAGGTACCCGTACGACGT-3′] and TgNup302 cassette R [5′-CCCACCGGCCGTTTCCAAGGTTTATCTACACACTGTCGCATTCCCAGTTAGCGGCCGCTCTAGAACTAGT-3′]. The selection was via chloramphenicol. TgNup503 overexpression line was attempted using CRISPR/CAS9 system where a cut was introduced at the ATG of the gene and a TUB8 promoter cassette flanked with 50 bp homology regions upstream and downstream of the ATG was inserted (TgNup503 gRNA OE F [5′-AAGTTAGAAGGATGGCAAGCTCCGGG-3′], TgNUp503 gRNA OE R [5′-AAAACCCGGAGCTTGCCATCCTTCTA-3′], TgNup503 OE cassette F [5′-CACTTTTCGCATTCCGGGCGGACGCCCTCCCTCGCTCCGTCGGCAGAAGGGGGCCCCCCCTCGACGGTATCGATA-3′], TgNup503 OE cassette [5′-GAGGTGGGCTGGCTTGAATTGCCAAACAACCCGCCACCGGAGCTTGCCATAAGAAAAAATGCCAACGAGTAGTTT-3′], Integration test F [5′-CCAAACGGTGTTACACAATCACC-3′], Integration test R [5′-CTTCCCCTGCAGGGGCACGAGTGCATCCCATG-3′], gDNA extraction test F [5′-CTTCGAGTACTTGCGGGGTC-3′], and gDNA extraction test R [5′-CCTGCTCTTCCTCCAGTGAC-3′]). Pools were tested once the parasites recovered from transfection. To create an inducible knockdown for TgTom40 (TGGT1_218280), CRISPR/Cas9-based promoter replacement strategy was employed (TgTom40 gRNA F [5′-AAGTTCGTCTTCATATTGCTTACGCG-3′], TgTom40 gRNA R [5′-AAAACGCGTAAGCAATATGAAGACGA-3′], TgTom40 cassette F [5′-GTAGTGTGTGTTGACTCTTCGTCGCTGCGAACTCGACCAGCGTAAGCAATAAGCTTCGCCAGGCTGTAAATCC-3′], TgTom40 cassette R [5′-CGAAATCCGGGCCGATGTTGCTGCTCTTCATGGCGAGAAGACGTCTTCATTGGTTGAAGACAGACGAAAGCAGTTG-3′], Integration test F [5′-GCAAACCGTAGTGTGTG-3′], Integration test R [5′-AGCCCGTTCCTCTATC-3′], DHFR R [5′-CACGGTTATCAAACCCGAG-3′], and T7S4 F [5′-CGGTTCGCTTGAAGAAGG-3′]). Guide RNA targeting the nearby sequence of the translation start codon was identified via the ChopChop tool (https://chopchop.cbu.uib.no/). The guide RNA sequence was cloned into the Cas9-YFP expressing plasmid with a U6 promoter (Tub-Cas9YFP-pU6-ccdB-tracrRNA) (Sidik et al., 2016). The endogenous 5′ sequence was replaced with a PCR product amplified from pDT7S4 (Sheiner et al., 2011) containing the ATc repressible T7S4 promoter and a DHFR selectable cassette through homologous recombination. Positive transfectants were selected using pyrimethamine and cloned via serial dilution.

### Transient overexpression of TgNup503 and TgTom40 fragments

TgNup503 and TgTom40 fragments were amplified from cDNA and cloned under TUB8 promoter into an overexpression plasmid (TgNup503_1 F [5′-CTTCCGAATTCATGGCAAGCTCCGG-3′], TgNup503_1 R [5′-CTTCCCCTGCAGGCACGAGTGCATCCCATG-3′], TgNup503_418 F [5′-CTTCCGAATTCATGGTGCTCTTTGGAGGTGC-3′], TgNup503_418 R [5′-CTTCCCCTGCAGGGGGACGGTGGGCG-3′], TgNup503_779 F [5′-CTTCCGAATTCATGTCGTCGCCGCGAAAC-3′], TgNup503_779 R [5′-CTTCATGCATCGTCGCCGGCCCC-3′], TgNup503_1297 F [5′-CTTCCGAATTCATGCATGAGTTCCTGGGCG-3′], TgNup503_1297 R [5′-CTTCCCTGCAGGGCTCGCGGCGAAG-3′], TgNup503_1557 F [5′-CTTCCCAATTGATGTGTGCCCACGGCG-3′], TgNup503_1557 R [5′-CTTCCATGCATCTGCTGCCTTCTTGTGAAG-3′], TgNup503_2073 F [5′-CTTCCCAATTGATGTGTCTGCGTCTTCGATTC-3′], TgNup503_2073 R [5′-CTTCATGCATCAGCTGCGTAGAGGTGAG-3′], TgNup503_2335 F [5′-CTTCCGAATTCATGCCGCTATACGCCACG-3′], TgNup503_2335 R [5′-CTTCATGCATCCGGAGCCCGAATGC-3′], TgNup503_2869 F [5′-CTTCCGAATTCATGCCGGCTTCAAGCAGAG-3′], TgNup503_2869 R [5′-CTTCATGCATCCGCCAGGGCAGTC-3′], TgNup503_3113 F [5′-CTTCCGAATTCATGTCCCTCCTCCGGTC-3′], TgNup503_3113 R [5′-CTTCATGCATCATCTGCTTCCGACGAAG-3′], TgNup503_3701 F [5′-CTTCCGAATTCATGACAGCCGTCGACCC-3′], TgNup503_3701 R [5′-CTTCATGCATCCGGAAACCGCGAGAG-3′], TgNup503_3891 F [5′-CTTCCGAATTCATGGTCTTCGAGGTGTGTCTG-3′], TgNup503_3891 R [5′-CTTCATGCATCTCAATTCTTGAGGTAGAAATAGATAAG-3′], TgTom40_83–155 F [5′-CTT​CCG​AAT​TCA​TGA​AAT​CCA​GCT​CCT​CCG​T-3′), and TgTom40_83–155 R [5′-CTTCATGCATCTGCCCTCGTTTTATCCAATT-3′]). Parasites were transfected with individual plasmids and fixed 24 h after transfection.

### Immunoprecipitation

For mass spectrometry and its validation via reciprocal IP: intracellular parasites (5 × 10^8^ tachyzoites) were purified via a 3-μm filter and washed twice with PBS. Cytoplasmic, nuclear, and insoluble extracts were prepared as described in Courjol et al. (2017). Pierce anti-HA or c-Myc tag beads (30-μl) were washed twice with 1x TBS (50 mM Tris-HCl, 150 mM NaCl, and 0.5 mM PMSF) and saturated with 500 µl of 1x TBS and 5 µg of BSA. Nuclear and insoluble extracts were added to the beads and incubated for 16–24 h at 4°C. The beads were washed five times with 1x TBS, Tween 20%, and 0.5 mM PMSF, and one time with 62.5 mM Tris pH 6.8 in the presence of PMSF. Finally, they were eluted with 1x DTT (0.5 MTris, pH 6.8, 20% SDS, saccharose, 1 M DTT), heated at 95°C for 5 min, and centrifuged at 14,000 rpm for 1 min. The supernatants were used for mass spectrometry or Western blot.

For TgTom40 interaction validation, 4 × 10^8^ to 2 × 10^9^ parasites were purified on a 3-μm filter, washed with PBS, resuspended in 1 ml 2% DDM/PBS/proteinase inhibitor, and incubated for 1 h at 4°C. Following 13,000 × *g* centrifugation for 15 min at 4°C, 2% of the supernatant was kept as the “input” sample and the remaining lysate was added to prewashed HA beads (88836; Pierce Anti-HA Magnetic Beads) and incubated for 16–24 h at 4°C. Beads were contained in a magnetic rack and 2% of the sample was then kept as “unbound” fraction. Beads were washed three times with 1 ml PBS/proteinase inhibitor and a last wash of 40 μl PBS/proteinase inhibitor, which was kept as the “last wash” sample. The beads were then resuspended in a loading dye (2% [wt/vol] SDS, 125 mm Tris–HCl pH 6.8, 10% [wt/vol] glycerol, 0.04% [vol/vol] β-mercaptoethanol, and 0.002% [wt/vol] bromophenol blue), boiled at 95°C for 5 min followed by cooling on ice and bead removal leaving “elute” fraction.

### Mass spectrometry proteomic analysis

Protein samples were separated on a 10% acrylamide SDS-PAGE gel, and five bands containing the whole sample (visualized by Coomassie Blue) were cut and sent to MS. An UltiMate 3000 RSLCnano System (Thermo Fisher Scientific) was used for separation of the protein digests. Peptides were automatically fractionated onto a commercial C18 reversed-phase column (75 µm × 150 mm, 2 µm particle, PepMap100 RSLC column, temperature 35°C; Thermo Fisher Scientific). Trapping was performed for 4 min at 5 μl/min with solvent A (98% H_2_O, 2% can, and 0.1% FA). Elution was performed using two solvents, A (0.1% FA in water) and B (0.1% FA in ACN) at a flow rate of 300 nl/min. Gradient separation was for 3 min at 5% B, 37 min from 5% B to 30% B, 5 min to 80% B, and maintained for 5 min. The column was equilibrated for 10 min with 5% buffer B prior to the next sample analysis. The eluted peptides from the C18 column were analyzed by Q-Exactive instruments (Thermo Fisher Scientific). The electrospray voltage was 1.9 kV and the capillary temperature was 275°C. Full MS scans were acquired in the Orbitrap mass analyzer over m/z 300–1,200 range with a resolution 35,000 (m/z 200). The target value was 5.00E+05. 10 most intense peaks with a charge state between 2 and 4 were fragmented in the HCD collision cell with a normalized collision energy of 27%, and a tandem mass spectrum was acquired in the Orbitrap mass analyzer with a resolution of 17,500 at m/z 200. The target value was 1.00E+05. The ion selection threshold was 5.0E+04 counts and the maximum allowed ion accumulation times were 250 ms for full MS scans and 100 ms for tandem mass spectrum. Dynamic exclusion was set to 30 s. Raw data collected during nanoLC-MS/MS analyses were processed and converted into *.mgf peak list format with Proteome Discoverer 1.4 (Thermo Fisher Scientific). MS/MS data were interpreted using search engine Mascot (version 2.4.0; Matrix Science) installed on a local server. Searches were performed with a tolerance on mass measurement of 0.2 D for precursor and 0.2 D for fragment ions, against a composite target decoy database (50,620 total entries) built with three strains of *T. gondii*, https://ToxoDB.org database (strains ME49, GT1, and VEG, release 12.0, September 2014, 25,264 entries) fused with the sequences of recombinant trypsin and a list of classical contaminants (46 entries). Cysteine carbamidomethylation, methionine oxidation, protein N-terminal acetylation, and cysteine propionamidation were searched as variable modifications. Up to one trypsin missed cleavage was allowed. For each sample, peptides were filtered out according to the cutoff set for protein hits with two or more peptides having more than 7 residues, ion score >25, identity score >0, and no false positive identification.

### Western blot

Parasite pellets were resuspended in a loading dye (2% [wt/vol] SDS, 125 mm Tris–HCl pH 6.8, 10% [wt/vol] glycerol, 0.04% [vol/vol] β-mercaptoethanol, and 0.002% [wt/vol] bromophenol blue), boiled at 95°C for 5 min, run on a precast gradient gel (Thermo Fisher Scientific) or 8% acrylamide gel, and transferred onto a nitrocellulose membrane (Protran) or PVDF. The membrane was blocked in 5% milk, PBS/0.2% Tween 20 for 20 min at RT (blocking buffer). Primary antibodies were added in a blocking buffer and incubated for 1 h at RT: myc (1:1,000; Cell Signalling), HA (1:1,000; Sigma-Aldrich), TgTom40 (1:1,000; gift from Giel van Dooren), TgMys ([Ovciarikova et al., 2017], 1:1,000), TgMic5 (1:1,000; gift from David Smith), CDPK1 (1:10,000; gift from Clare Harding), TgENO2 ([Courjol et al., 2017], 1:1,000). The membrane was then washed three times with PBS/0.2% Tween. Secondary antibodies were added in a blocking buffer and incubated for 1 h at RT: IRDye 800CW, 680RD (1:10,000; LIC-COR). Incubation was followed by three washes with PBS/0.2% Tween. The signal was detected and quantified with Odyssey CLx or the signal was revealed using the Western Pico SuperSignal substrate.

### RT-qPCR

Parasites were cultured with or without 0.5 µg/ml ATc for 24 h, purified on a 3-μm filter, and washed once with PBS. RNA was extracted with RNeasykit (Qiagen). cDNA was made using High Capacity RNA-to-cDNA Kit (Applied Biosystems) (TgNup503 F [5′-CTTCGAGTACTTGCGGGGTC-3′], TgNup503 R [5′-AGCGTGCCATCTCGGCGC-3′]), TgActin F ([5′-GGGACGACATGGAGAAAATC-3′], and TgActin R [5′-AGAAAGAACGGCCTGGATAG-3′]). qRT-PCR was set up with 10 ng of cDNA in technical triplicates alongside water only and RNA only controls. qRT-PCR was run in 7,500 Real Time PCR system (Applied Biosystem). Expression was assessed with the 2^−ΔΔCt^ method (Livak and Schmittgen 2001).

### Growth assays

Plaque assays were performed using 6-well plates containing human fibroblast cells infected with 200 parasites per well in media with or without 1 µg/ml ATc, fixed after 7 days of infection, and labeled with a crystal violet solution.

For replication assay, parasites were seeded onto HFFs grown on glass coverslips and spun gently (1–3 min at 100–300 × *g*) to allow the parasites to land on the host cells. They were then cultured with or without 0.5 µg/ml ATc for 16 hr/24 h followed by fixation and immunofluorescent assay. Single and dividing parasites were omitted from counting.

### tRNA pull-down

5 × 10^8^ parasites were resuspended in 1 ml lysis buffer (50 mM HEPES-KOH, pH 7.4, 210 mM mannitol, 70 mM sucrose, 1 mM EGTA, 5 mM EDTA, 10 mM KCl, 1 mM DTT, and 1 protease inhibitor cocktail tablet [Complete Mini, EDTA-free; Roche] per 50 ml). Lysis was achieved via nitrogen cavitation as previously described (Maclean et al., 2021). Unbroken parasites were spun out at 1,500 × *g*. The cleared lysate was spun down at 16,000 × *g* for 25 min and pellets were used for IP. 50 μl of paramagnetic streptavidin resin (DYNAL Magnetic Beads; Invitrogen) was washed three times in 500 μl of 1X SSC buffer (150 mM NaCl, 15 mM tri-sodium citrate dihydrate, pH 7.2). The washed resin was resuspended in 500 μl 0.5x SSC and synthetic 5′ biotinylated *T. gondii* tRNA^Ile^ or tRNA^Met-i^ (Dharmacon) was added to a final concentration of 2 μM and incubated at 65°C for 10 min to form the tRNA-streptavidin affinity resin. The tRNA-bound resin was washed three times with 300 μl of 0.1x SSC and equilibrated in 300 μl of protein binding buffer (PB) (160 mM MOPS, 310 mM sucrose, 6.25 mM MgCl_2_, 100 mM KCl, 9 mM DTT, 2 U/300 μl of RNAse inhibitor [10 U/μl stock], 1 mg/ml BSA). Each 16,000 × *g* pellet was adjusted to 6.0 × 10^9^ per 300 μl PB buffer containing 1% Triton X-100, and this crude-mitochondria lysate replaced the PB onto the beads, incubated for 30 min at RT. Unbound proteins were collected and the tRNA affinity resin was washed six times in 500 μl of PB buffer. tRNA-bound proteins were eluted in 50 μl of elution buffers containing increasing ionic concentrations of NaCl (0.25, 0.5, 0.75, 1 M NaCl; and 20 mM MOPS, pH 7.2). Protein samples from each elution were TCA precipitated and separated on 8–18% SDS-PAGE. Acrylamide gels were stained with SYPROTM Orange Protein Gel Stain, silver staining, or Coomassie blue. For LC-MS/MS analysis, bands of interest were cut out of the gel and sent to Polyomics, Glasgow, and analyzed as previously described (Maclean et al., 2021).

### Northern blot

Mitochondrial tRNAs were analyzed as described previously (Esseiva et al., 2004). 2 × 10^8^ parasites were filtered via a 3-μm filter, washed once with 1x PBS, and resuspended in 0.05% digitonin in SoTE (0.6 M sorbitol, 20 mM Tris–HCl, pH 7.5, and 2 mM EDTA). Cytosolic RNA was degraded using RNase A (1 mg/ml) and a “crude mitochondria” pellet was obtained by centrifugation. The crude mitochondrial fractions were suspended in TRIzol and RNA was isolated using the acid guanidium method (Chomczynski and Sacchi, 1987). RNA pellet was resuspended in 2x RNA loading dye (catalog nr. R0641; Thermo Fisher Scientific), separated on a 10% polyacrylamide gel containing 8 M urea, and transferred onto a nylon membrane followed by UV irradiation. Radioactive probes against the tRNAs and rRNAs were made by labeling complement oligos with [γ-32P]ATP using polynucleotide forward kinasing reaction (tRNA-Ile [5′-TGGTCCCAACCGGGATCG-3′] and mitoLSU rRNA [5′-GACAAGGATTTTCCTACCTT-3′]). Blots were hybridized with the radiolabeled probe overnight in the Invitrogen hybridization buffer for Northern blots (Prehybridization/Hybridization Buffer; catalog nr. AM8677; Invitrogen NorthernMax). The radioactive signal was detected using Typhoon FLA 9500 phosphorimager and quantified using ImageJ.

### Complex IV and V assays

Mitochondrial complex IV and V were assayed as previously described (Lacombe et al., 2019; Maclean et al., 2021). The parasite pellet was resuspended in Clear Native solubilization buffer (50 mM NaCl, 2 mM 6-aminohexanoic acid, 50 mM imidazole, 2% (wt/vol) n-dodecylmaltoside, 1 mM EDTA–HCl pH 7.0) for 10 min and spun down (16,000 × g; 4°C). The supernatant was mixed with glycerol and ponceau and loaded onto a precast 4–16% Bis-Tris Polyacrylamide Native gel (4–16%, Bis-Tris, 1.0 mm, Mini Protein Gels; NativePAGE). Complex IV activity was visualized in-gel using cytochrome C and 3,3′-diaminobenzidine tetrahydrochloride (DAB) as substrates in the oxidation buffer (1 mg ml−1 cytochrome c, 50 mM KH_2_PO_4_, pH 7.2, 0.1% (wt/vol) 3,3′-diaminobenzidine tetrahydrochloride) incubated for 2 h. Complex V assembly was analyzed by immunoblotting using anti-ATP Synthase β subunit antibody.

### Mitochondrial membrane potential

Intracellular parasites were washed in their host with 3 ml warm complete media and then recovered by scraping host cells, passing through a 26G needle and through a 3-μm filter. Parasites were centrifuged at 1,500 × *g* for 10 min and the pellet was resuspended in 1 ml media with 5 µM JC-1 (Thermo Fisher Scientific) dye and incubated at 37°C for 30 min. Parasites were centrifuged again, resuspended in a FACS Buffer (1x PBS, 1% BSA, 1 mM EDTA) at a concentration of 10^6^–10^7^ parasites/ml, and analyzed on a FACS Celesta. Parasites were gated using forward and side scatters (with mNeon-Green expressing parasites used as a control). Membrane potential was analyzed by comparing JC-1 monomer positive cells (Blue 488 nm laser and FITC filter) versus JC-1 aggregate positive cells (Y-G 535 nm laser and PE filter set). As a positive control for membrane potential disruption, a subset of parasites was incubated with 1 µM Valinomycin for 30 min immediately prior to staining.

### ROS level

Intracellular parasites were washed with 3 ml warm Hanks’ balanced salt solution (HBBS; 14025092; Thermo Fisher Scientific), and intracellular parasites were recovered by scraping host cells, passing through 26G needle, and passing through 3 μm filter. Parasites were centrifuged at 1,500 *g* for 10 min, the pellet was resuspended in 1 ml 500 nM MitoSOX Red (M36008; Thermo Fisher Scientific), and incubated at 37°C for 30 min then centrifuged as before. Parasites were finally resuspended in HBBS to a concentration of 10^6^–10^7^ parasites/ml and analyzed on a FACS Canto II (BD Biosciences). Parasites were gated as above, and the MitoSOX signal was measured using the 488-mm Blue laser and PE filter set. For each sample, 100,000 events were recorded.

### Extracellular flux analysis

Oxygen consumption rate (OCR) and extracellular acidification rate (ECAR) were measured using a Seahorse XF HS Mini Analyser, as described assay (Hayward et al., 2022). Briefly, parasites were incubated in the presence or absence of ATc for 24 h before harvest. Fully lysed parasites were passed through a 3-μm polycarbonate filter and washed with Seahorse XF DMEM base medium supplemented with 5 mM glucose and 1 mM glutamine. Cells were counted and resuspended in a supplemented base medium. 1.5 × 10^6^ parasites were added per well to a plate treated with poly-L-lysine, before centrifugation at 800 × *g*. Parasite OCR and ECAR were then measured using the Seahorse XF HS Mini Analyser. Basal OCR was calculated by taking the initial OCR reading and subtracting the OCR after the addition of 1 μM atovaquone. Three independent experiments were performed.

### Statistical analysis

Experiments were carried out in biological replicates to a total *n* as stated in figure legends. Normality was assumed but not formally tested, and for heavily skewed data, non-parametric tests were used. Data were plotted and analyzed in GraphPad Prism 9, the nature of testing is stated in the figure legend and/or main text. P < 0.05 is displayed in the graphs, NS is omitted.

### Figures

Figures were assembled in Inkscape: Inkscape Project. (2020). *Inkscape*. Retrieved from https://inkscape.org.

### Online supplemental material

Fig. S1 shows the super-resolution microscopy analysis of nuclear pore and outer mitochondrial membrane markers. Fig. S2 shows the immunofluorescence of two hypothetical proteins found in the pull-down of TgNup503. Fig. S3 attempts to express fragments of TgNup503 or TgTom40, or to overexpress TgNup503, resulting in the localization a TgTOM40 fragment to the nuclear periphery. Fig. S4 shows genetic manipulations of TgNup503, attempts for overexpression, and the generation and analysis of inducible knock-down. Table S1 proteomics results from pull-down of TgNup503. Table S2 proteomics results from tRNA pull-down. Videos 1 and 2 are two parallel movies of the tomography of the mitochondrial nucleus membrane contact site around the nuclear pore.

## Supplementary Material

Table S1 shows proteomics results from pull-down of TgNup503.

Table S2 shows proteomics results from tRNA pull-down.

SourceData F3 is the source file for Fig. 3.

SourceData F6 is the source file for Fig. 6.

SourceData FS1 is the source file for Fig. S1.

SourceData FS4 is the source file for Fig. S4.
