# Supplementary material for: Two ancient membrane pores mediate mitochondrial-nucleus membrane contact sites
Source: J Cell Biol. 2024 Mar 8;223(4):e202304075. doi: 10.1083/jcb.202304075 (PMC10923651; doi:10.1083/jcb.202304075)

Figure 3A, membrane cut prior to antibody probing

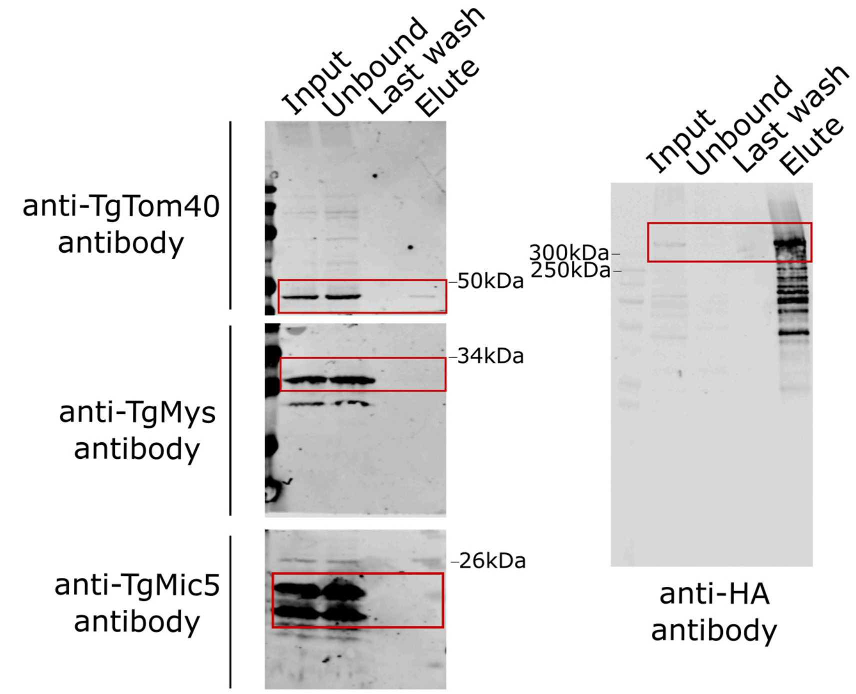

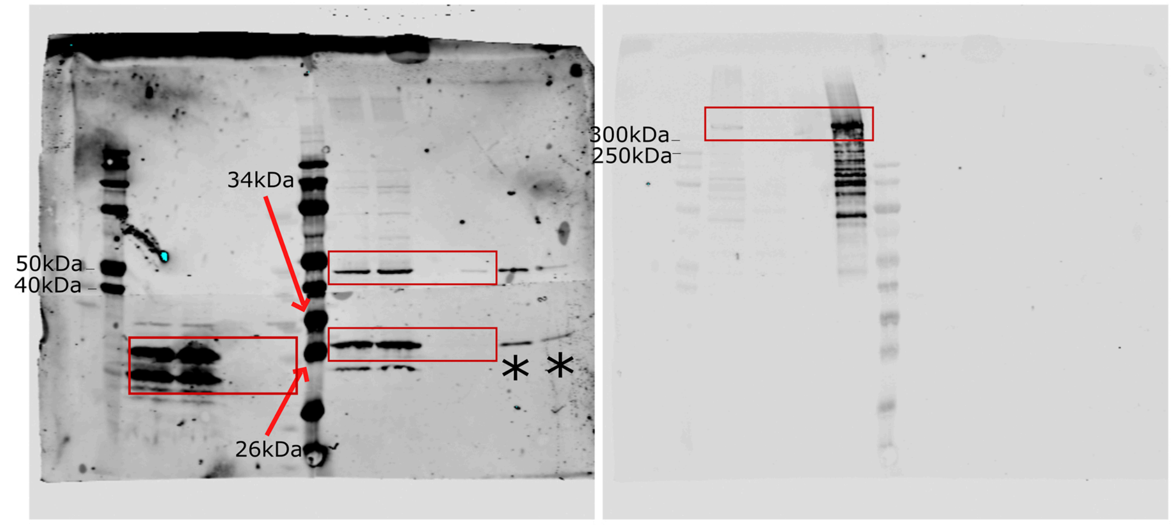

* lane with either a repetition or an unrelated sample

Figure 3B

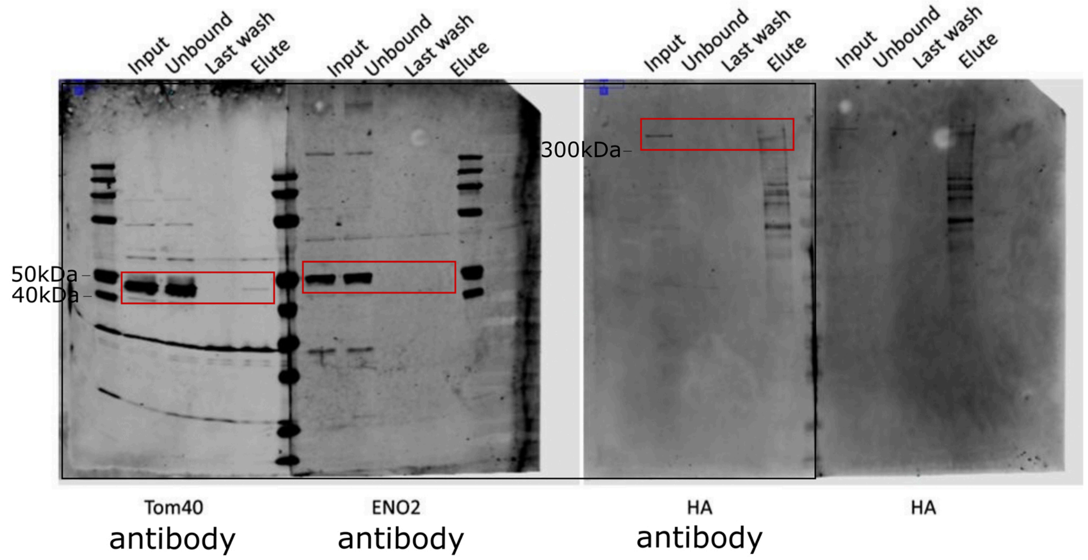

Figure 3C, membrane cut prior to antibody probing

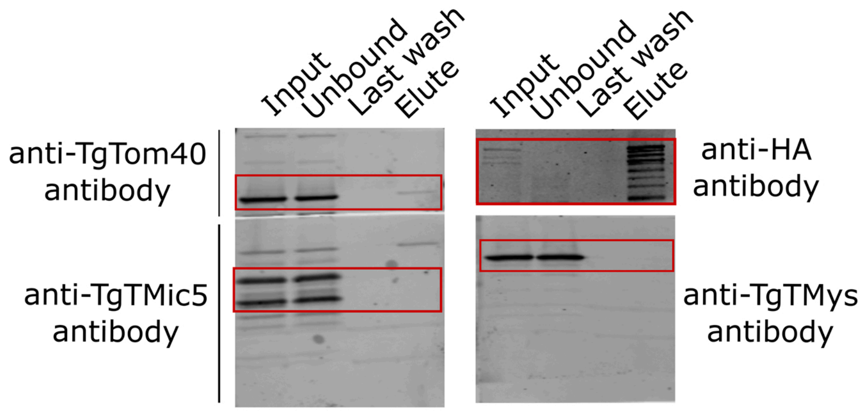

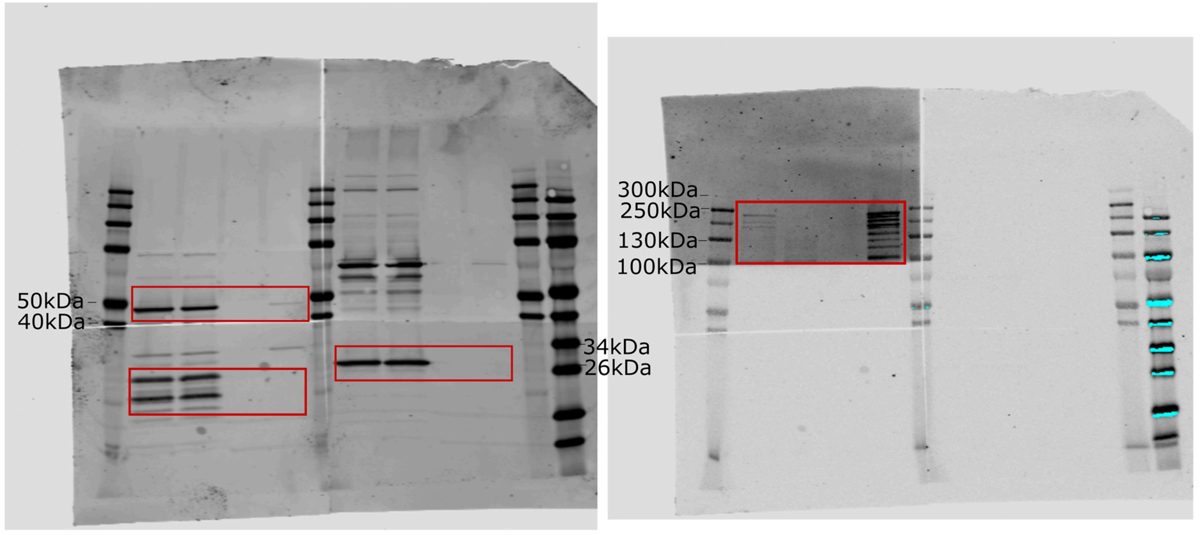

Supplement: SourceData F3 — is the source file for Fig. 3. [file JCB_202304075_SourceDataF3.pdf]
