# Supplementary figures and images for: Two ancient membrane pores mediate mitochondrial-nucleus membrane contact sites
Source: J Cell Biol. 2024 Mar 8;223(4):e202304075. doi: 10.1083/jcb.202304075 (PMC10923651; doi:10.1083/jcb.202304075)

Figure 6A

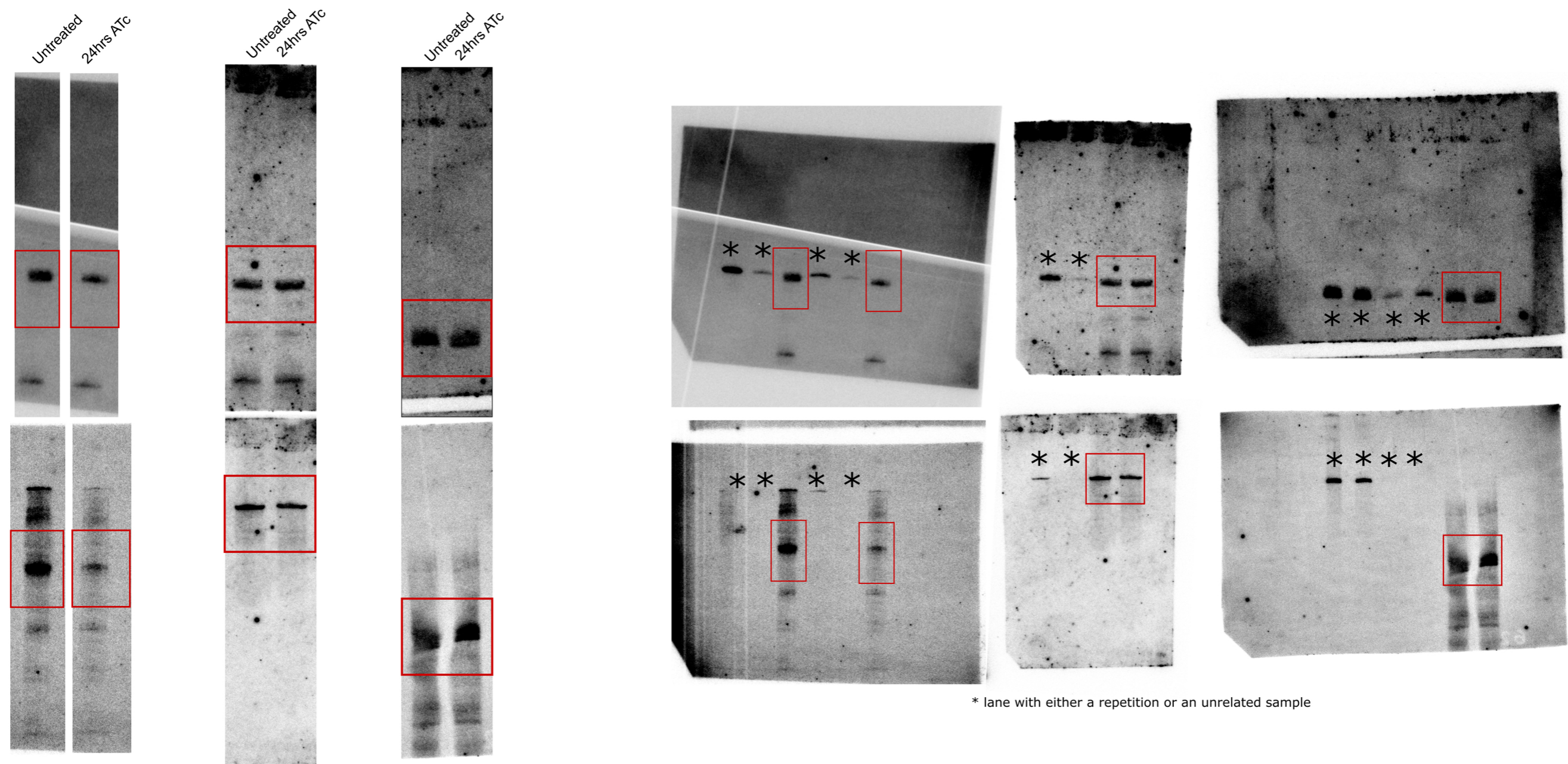

Figure 6B

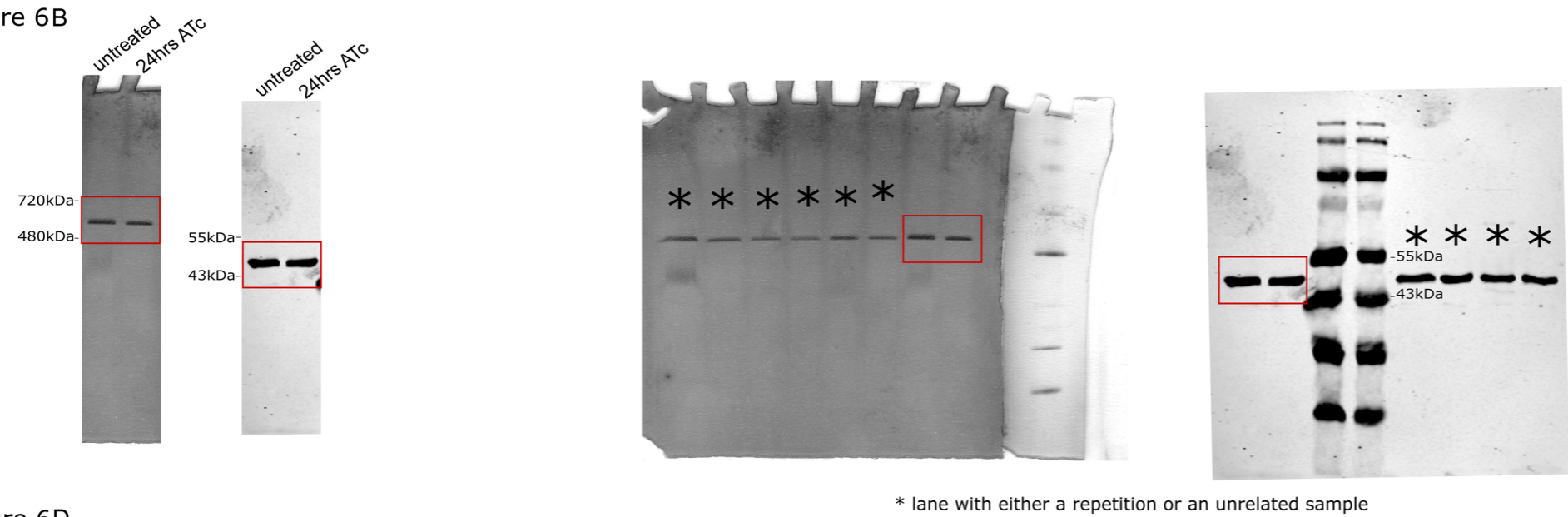

Figure 6D

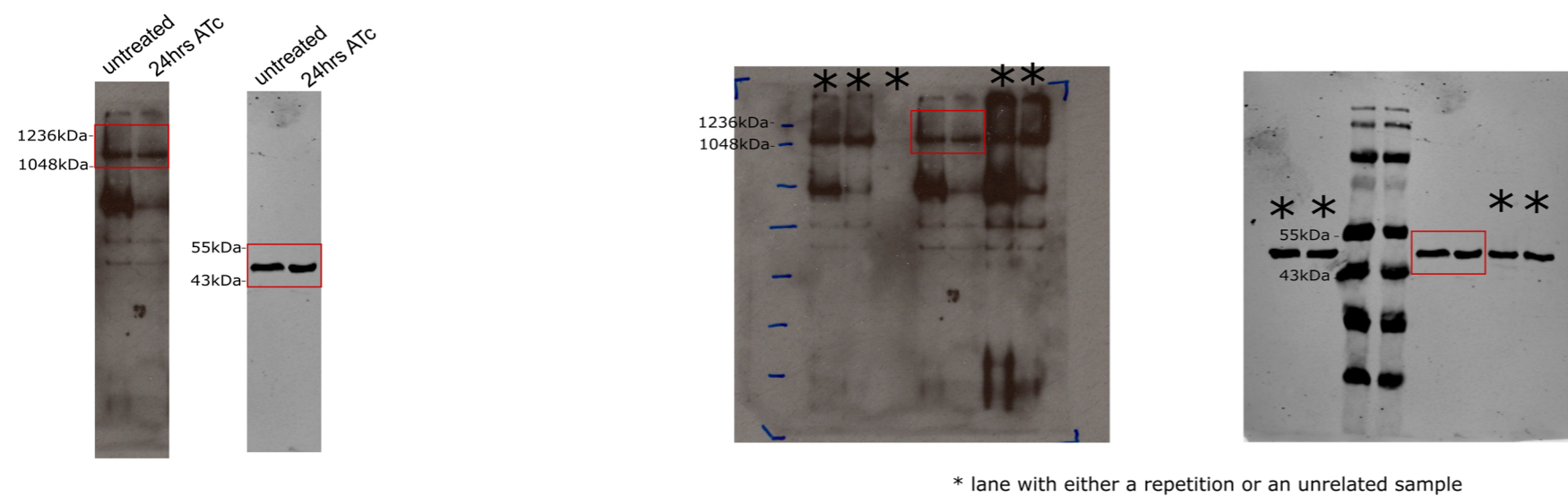

Supplement: SourceData F6 — is the source file for Fig. 6. [file JCB_202304075_SourceDataF6.pdf]

Figure S1N

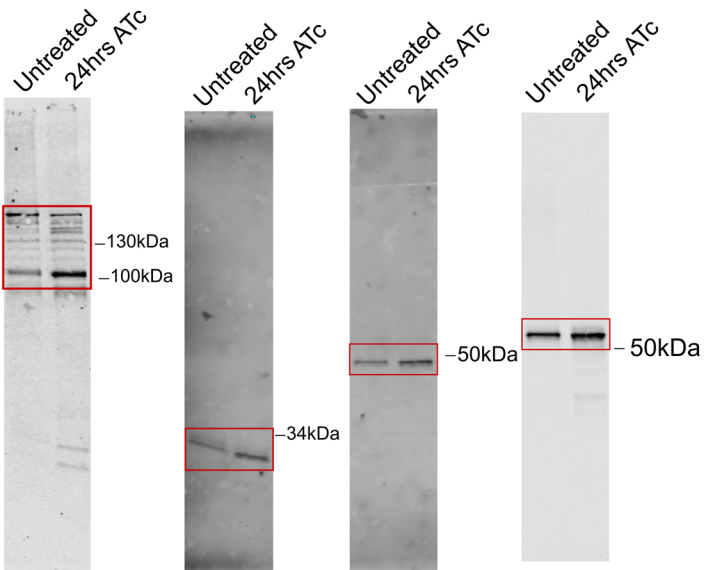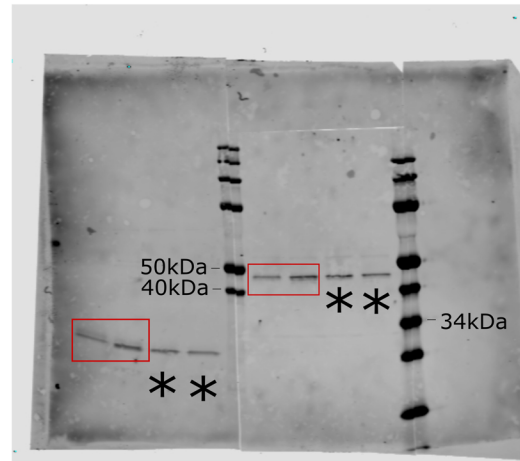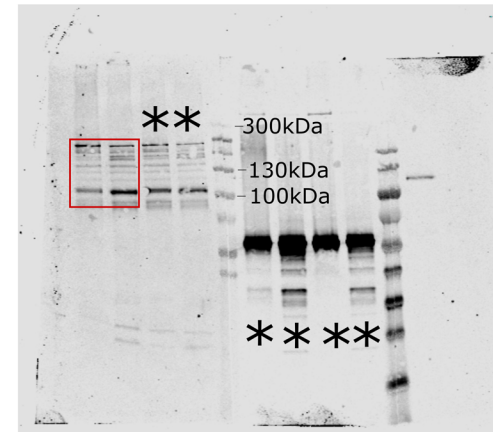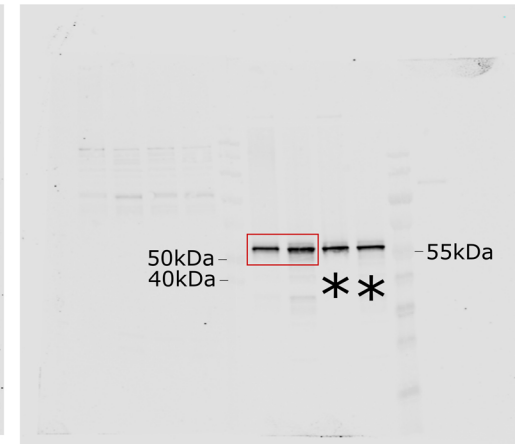

\* lane with either a repetition or an unrelated sample

Supplement: SourceData FS1 — is the source file for Fig. S1. [file JCB_202304075_SourceDataFS1.pdf]
