# Supplementary material for: Two ancient membrane pores mediate mitochondrial-nucleus membrane contact sites
Source: J Cell Biol. 2024 Mar 8;223(4):e202304075. doi: 10.1083/jcb.202304075 (PMC10923651; doi:10.1083/jcb.202304075)
Supplement: SourceData FS4 — is the source file for Fig. S4. [file JCB_202304075_SourceDataFS4.pdf]

Figure S4B

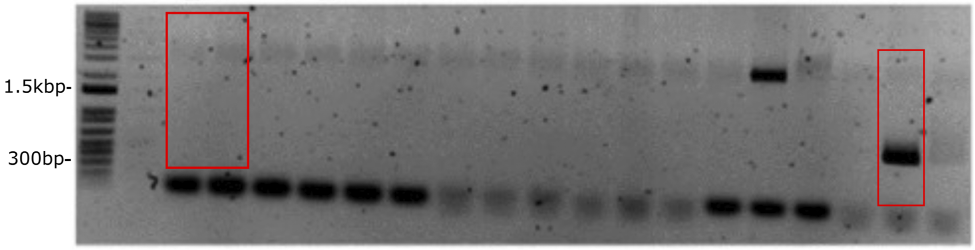

FigureS4D

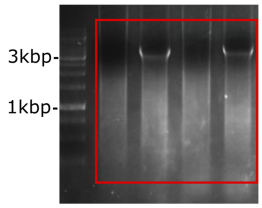

* lane with either a repetition or an unrelated sample

Figure S4F

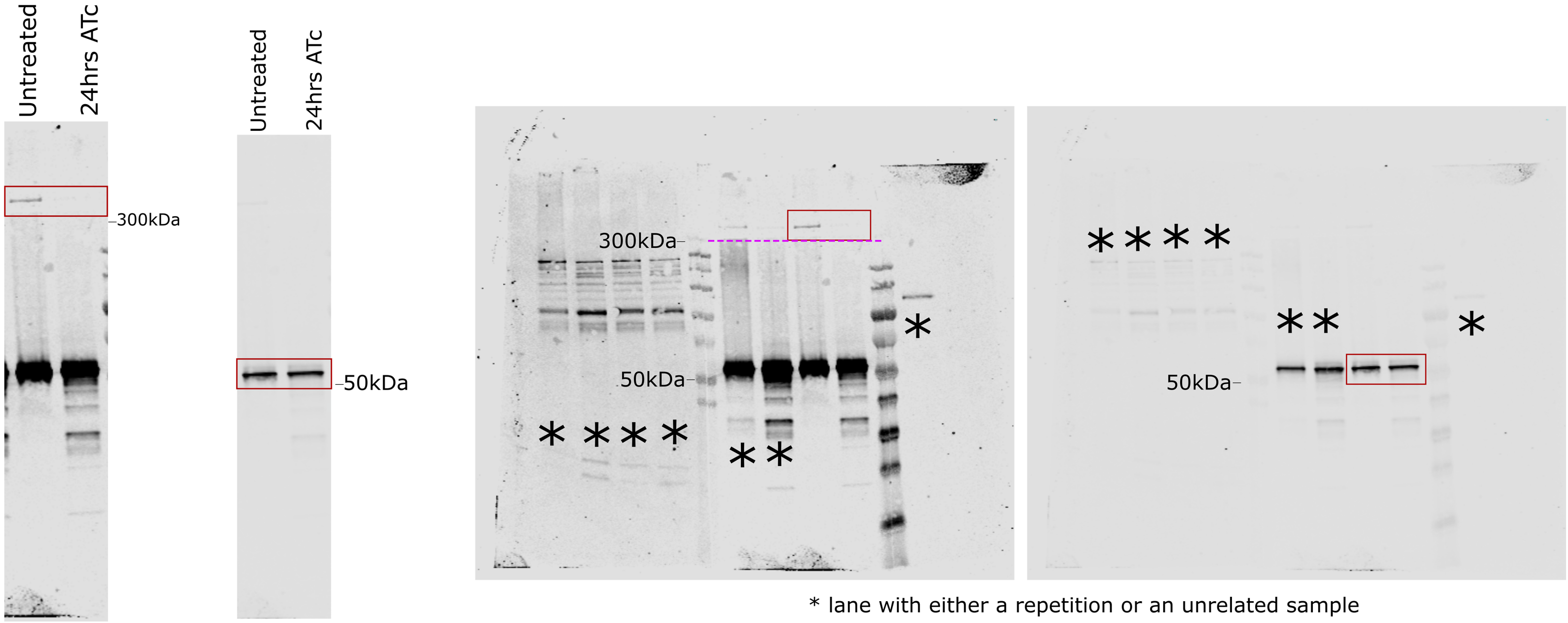

* lane with either a repetition or an unrelated sample
-------- Membrane cut horizontally priot to probing

Figure S4K

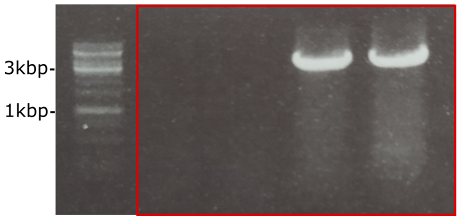

Figure S4L

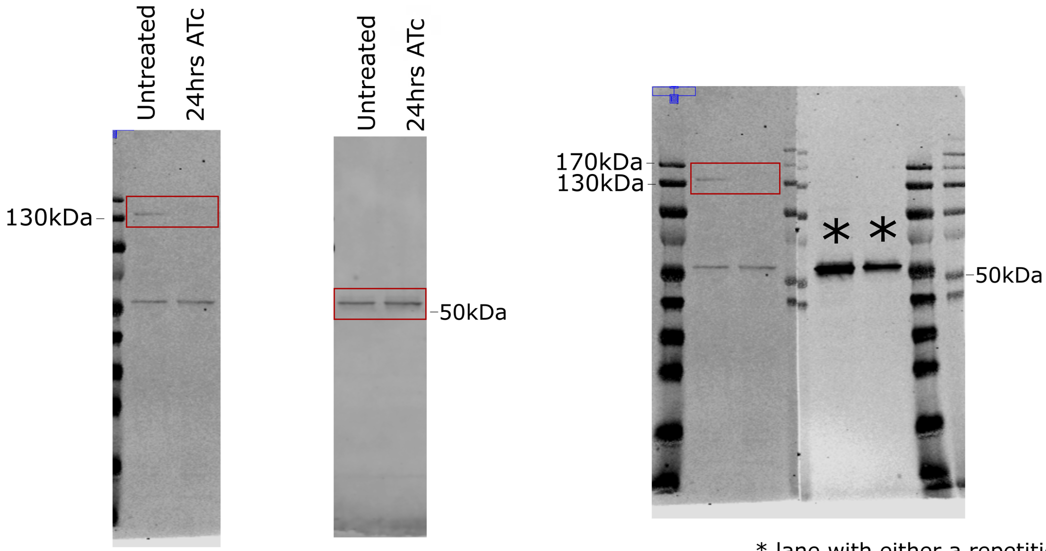

* lane with either a repetition or an unrelated sample

Figure S4N

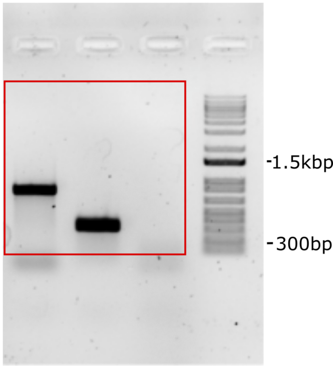

Figure S6O

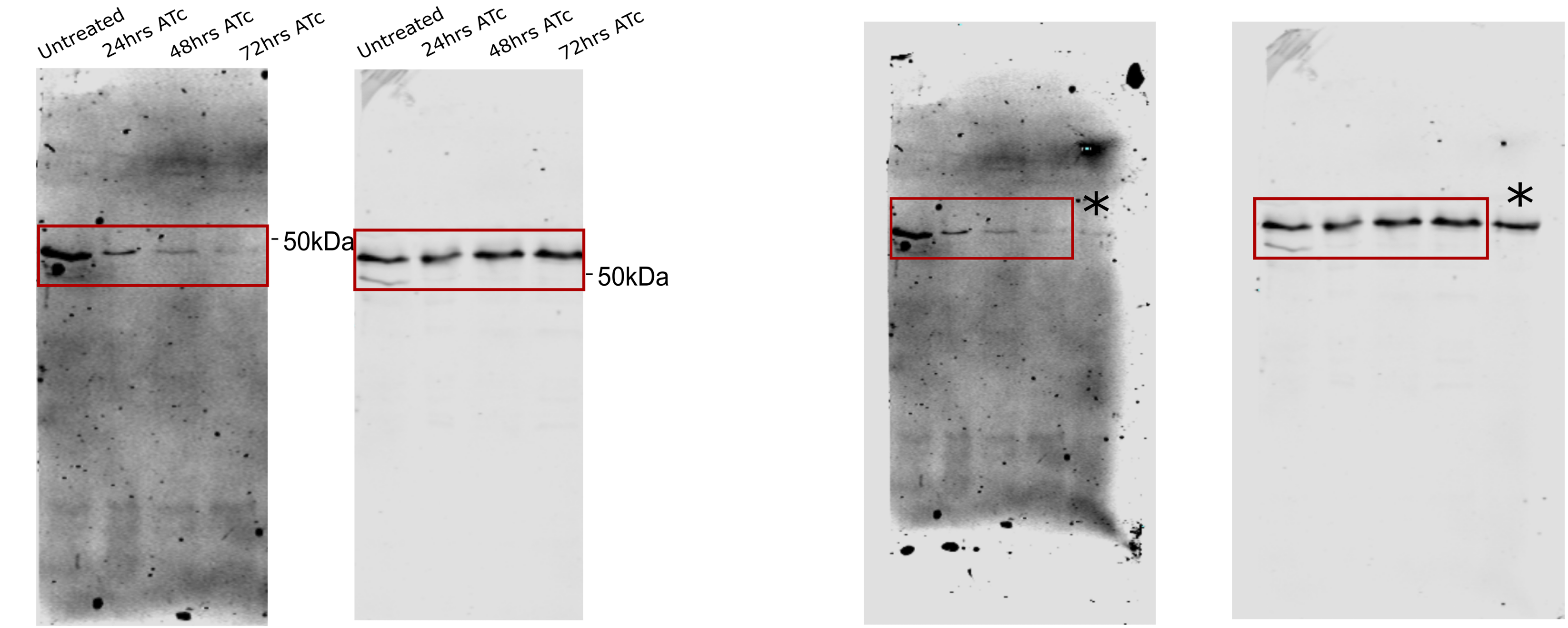

* lane with either a repetition or an unrelated sample

Figure S6E

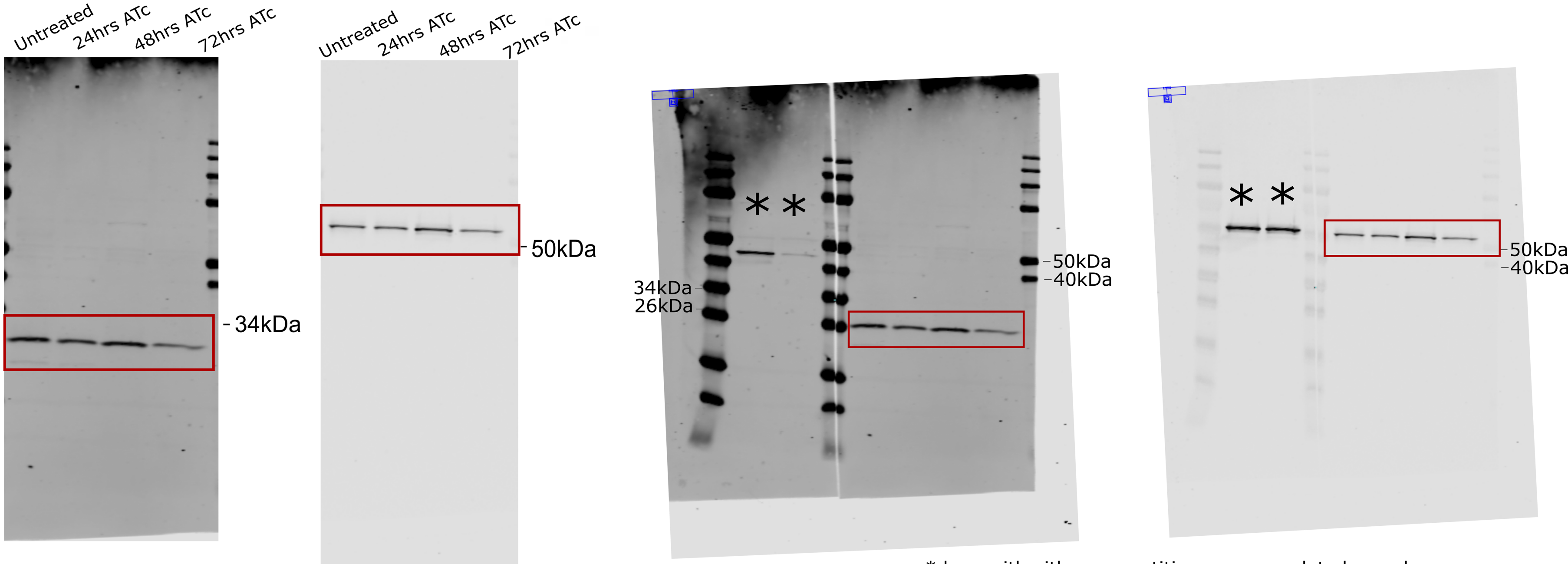

* lane with either a repetition or an unrelated sample
